# Investigation on the unsteady aerodynamic coefficients of iced conductors and the applicability of quasi-static assumptions

**DOI:** 10.1038/s41598-026-51439-5

**Published:** 2026-06-21

**Authors:** Guanghui Liu, Zhongbin Lv, Bo Zhang, Chuan Wu, Yuhang Nie, Haobo Liang

**Affiliations:** 1 State Grid Henan Electric Power Research Institute, Zhengzhou, 450052 People’s Republic of China; 2 Chongqing Jiaotong university, Chongqing, 400074 PR China; 3 Central South University, Hunan, 410083 PR China

**Keywords:** Unsteady aerodynamic coefficients, wind tunnel tests, Quasi-static assumptions, Hysteresis loops, BP neural network, Engineering, Physics

## Abstract

This study investigates the aerodynamic characteristics of crescent-shaped iced conductors through combined wind tunnel experiments and numerical analysis. Wind tunnel tests were conducted to obtain both steady and unsteady aerodynamic coefficients, the latter measured using an oscillating conductor model. The steady coefficients were acquired under various wind velocities ranging from 10 to 18 m/s and ice thicknesses of 14, 24 and 33 mm, covering trans-critical and super-critical Reynolds number regimes. Analytical expressions relating steady coefficients to the angle of attack were derived via polynomial fitting. A BP neural network model was subsequently developed and trained on this experimental data to predict aerodynamic coefficients for untested conditions. Furthermore, a MATLAB-based quasi-static model was established to convert steady coefficients into unsteady equivalents, enabling a direct comparison with the unsteady oscillation test data to validate the quasi-static assumption. The results confirm that the quasi-static assumption retains reasonable applicability in the studied regime. More importantly, the developed BP neural network demonstrates robust predictive capability, with its outputs showing close agreement with wind tunnel measurements, achieving determination coefficients exceeding 0.95. This work provides enhanced analytical tools and validated data for improving the prediction of conductor galloping—a critical wind-induced vibration—thereby contributing to the safety assessment and structural design of overhead transmission lines.

**Supplementary Information:**

The online version contains supplementary material available at https://doi.org/10.1038/s41598-026-51439-5.

## Introduction

High-voltage transmission lines are an important part of power facilities, and their damage will cause major economic losses. Therefore, the safe operation of transmission lines has attracted widespread attention when the conductors are covered with heavy ice. However, because of the complexity and instability of the natural environment, when the climate reaches certain specific conditions, the transmission lines may be covered with ice on its windward side, which makes the cross section of the conductor become asymmetrical, resulting in aerodynamic forces acting on the conductor unstable. Under a certain wind velocity and angle of attack, the conductor is prone to galloping. Galloping is a low-frequency, large-amplitude self-excited vibration of transmission lines. Because of the large amplitude and long duration of conductors galloping, this phenomenon can cause damage to poles and towers, lines, overhead power line fittings and other components, and serious accidents such as line tripping, breakage of conductors and collapse of towers, which poses great harm to the power system. Therefore, studying the galloping characteristics of iced conductors is of great significance to the development of anti-galloping technology for transmission lines.

The aerodynamic forces, namely the lift, drag and torsional moment, acting on the iced conductors with non-circular section in wind field play key role in the inducing of galloping of transmission lines^1^. Therefore, in order to investigate the galloping characteristics of the iced conductors, its aerodynamic characteristics must first be determined. At present, it is a relatively direct and common method to obtain the aerodynamic three-component force coefficients of conductors through wind tunnel test. Den Hartog^2^ first obtained the aerodynamic coefficients of different types of iced conductor models. They pointed out that the aerodynamic coefficients determined by the quasi-steady-state wind tunnel test is the basis for the investigation of conductors galloping in uniform and turbulent flows. Based on the result of this research, scholars generally use quasi-static assumptions when studying the galloping behavior of iced conductors, which is to use the aerodynamic coefficients obtained in steady tests for the calculation of dynamic galloping^3–5^.

Since the aerodynamic forces of the conductors are constantly changing with the conductor position, speed and other factors when the conductor is galloping, this “quasi-static” assumption is not completely applicable. The pitching motion mechanism and the protective measures of galloping were proposed by Nigol et al.^6–8^ through the experimental investigation of iced single conductors. Chabart and Lilien^9^ measured the steady aerodynamic characteristics of single conductors by using the wind tunnel test. Lou et al.^10^ carried out wind tunnel tests, obtained the aerodynamic coefficients of single and bundle conductors with different ice thicknesses, and determined the range of wind attack angles to galloping. Similarly, their experimental investigations have focused on the stable aerodynamic coefficients of the iced conductors and have not conducted detailed investigations on the accuracy of the quasi-static theory (QST). In the investigation of unsteady aerodynamics, Kimura et al.^11,12^ conducted experimental investigation on the lift and moment coefficients of similar crescent-shaped iced conductors under lateral and pitching vibrations, and the applicability of steady aerodynamic coefficients is verified to a certain extent, and the difference between unsteady aerodynamics and **s**teady aerodynamics is compared. Li^13^ studied the aerodynamic characteristics of two types of iced conductors by means of quasi-steady and dynamic wind tunnel tests and discussed the applicability of the quasi-static hypothesis in galloping analysis of iced conductors. However, at present, there is still a lack of more, and more in-depth investigation on the unsteady aerodynamic forces of iced conductors and the applicability of the quasi-static assumptions.

On the other hand, wind tunnel tests have the disadvantages of long cycle and high cost. The numerical simulation method based on computational fluid dynamics (CFD) can not only make up for these shortcomings, but also has the advantages of investigating the fine structure and development process of the flow, simulating complex flow fields, etc., so it has gradually become an effective means to identify the aerodynamic parameters of the conductors^14^. However, there is a certain deviation between the results of the numerical simulation and the wind tunnel test^15^. In order to achieve sufficient numerical simulation accuracy, a very fine model grid is required, which leads to lower efficiency and higher calculation costs.

Therefore, in recent years, the application of artificial neural network methods in the fields of fluid dynamics and structural wind resistance has begun to attract much attention^16^. Based on the BP neural network and deep learning methods, the prediction models for aerodynamic characteristics can be established to map the complicated nonlinear relations between the aerodynamic coefficients and the parameters of structure. Dumitru et al.^17^ used feedforward neural network to simulate and train wind power generation data. This method improves the prediction performance of wind power and verifies the feasibility of the neural network method. Wang et al.^18^ used K-means clustering and deep belief networks (DBN) to predict short-term wind energy, and compared with the prediction results of BP neural network and Morlet wavelet neural network, its prediction accuracy is significantly improved. Jin et al.^19^ established a model of the relationship between the cylindrical pressure and the velocity field by using a convolutional neural network (CNN), with this model, only need to measure the pressure distribution on the bluff body that will predict the structure wake velocity field. Chen et al.^20^ established a neural network model of aerodynamic parameters of the main girder of a long-span bridge through artificial neural network technology, and the results showed that the error between the neural network output and the expected output meets the expected requirements. Based on the convolutional neural network (CNN), Chen et al.^21^ presented a graphical model, in which the transformed aerofoil image was set as input, and the model can predict the pitch-moment, lift and drag coefficients with high accuracy. From the research of the above-mentioned scholars, it can be seen that the practicality of neural network prediction in reality, and it also provides a new way to obtain aerodynamic coefficients of iced transmission lines.

In this paper, the steady and unsteady aerodynamic coefficients of the conductors are obtained through wind tunnel tests firstly, and the unsteady aerodynamic coefficients are deeply analyzed. Based on the sample data of steady aerodynamic coefficients and the quasi-static assumption, the conversion of steady aerodynamic coefficients to unsteady aerodynamic coefficients is realized by MATLAB, which is compared with the unsteady aerodynamic coefficients obtained from wind tunnel tests, and the applicability of quasi-static assumption is discussed. The steady aerodynamic coefficients are processed by polynomial fitting methods commonly used by scholars, and after converting them into unsteady aerodynamic coefficients, the amount of work done by the aerodynamic force under different fitting orders is analyzed, and the appropriate fitting order is determined. Then, based on the BP neural network method, the aerodynamic characteristics prediction model is established, and the aerodynamic coefficients of the transmission line at different ice thickness and different wind velocities are predicted, and the results are compared with the wind tunnel test results to verify the feasibility of the BP neural network method. Finally, the prediction results are also used as the basic data and converted into unsteady aerodynamic coefficients, which are compared with the unsteady aerodynamic coefficients obtained by the above-mentioned different processing methods and the wind tunnel test results.

## Wind tunnel tests

### Test model

**Fig. 1:**
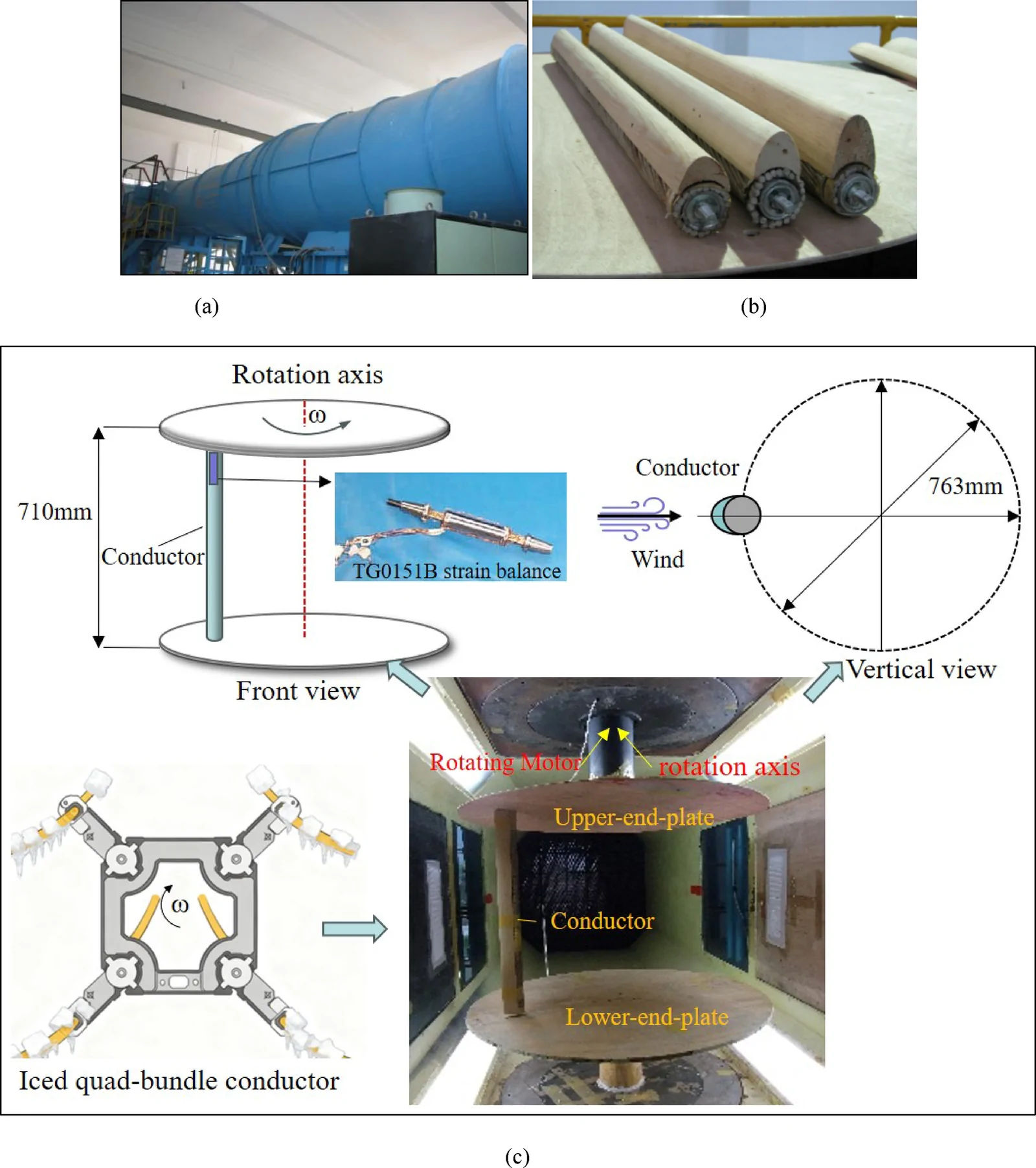
Wind tunnel tests for the iced conductors. (**a**) Wind tunnel; (**b**) Iced conductor models; (**c**) Schematic of the experimental setup.

The experiments were conducted in the 1.4 m×1.4 m open-return wind tunnel (Fig. 1a) at the Low Speed Aerodynamics Institute of the China Aerodynamics Research and Development Center (CARDC). The wind velocity was continuously adjustable within the range of 0 to 65 m/s, with a turbulence intensity of ≤ 0.12% in the test section. The model of the test conductor is 4XLGJ-400/50 steel core aluminum stranded conductor, and the diameter of the bare conductor is 27.6 mm. For the wind tunnel tests, the model diameter was scaled to 33 mm (scaling ratio 1:1.2) to accommodate the internal strain balance. This diameter falls within the typical range of high-voltage transmission lines. The outer surface of the conductor model is wrapped with rubber tubes to simulate the actual conductor shape. Field observations, such as the classification of 124 cases by M. Masataka^22^, demonstrate that despite the variety of ice shapes, crescent-shaped ice appears in a considerable proportion of actual galloping events. Therefore, this shape represents a critical case for investigation. At the same time, the crescent shape is a typical and easy to quantify ice type. Therefore, this experiment mainly focuses on the aerodynamic coefficients of the crescent-shaped iced conductors. According to the actual situation, the ice thickness is mainly 14 mm, 24 mm and 33 mm. The artificial crescent-shaped ice is made of light wood with a density close to real ice (836.81 kg/m^3^), as shown in Fig. 1b.

The experimental configuration employed in this study is schematically depicted in Fig. 1c. Field observations indicate that galloping of iced quad-bundled conductors often involves rotational motion. To investigate the influence of this rotational effect on aerodynamic parameters and to evaluate the validity of the quasi-static assumption commonly used in engineering practice, an experiment was designed featuring a single sub-conductor rotating about the center of the quad-bundle. This setup effectively isolates the rotational effect from other potential interference factors. The rotation is driven by an electric motor, with the sub-conductor tracing a circular path with a diameter of 763 mm. It is important to note that, given the eccentricity of the conductor’s position relative to the rotation axis, the motion experienced by the conductor is not simple pitching; rather, it is a combination of pitching and plunging motion. To be specific, a high-precision servo motor system was used to generate the prescribed torsional oscillations. The dynamic motion, following a cosine law at frequencies of 0.1, 0.2, and 0.33 Hz, was implemented via a programmable motion controller. A closed-loop control strategy, utilizing real-time feedback from an optical encoder on the motor shaft, ensured high-fidelity tracking of the target trajectory. This system maintained precise control over both the amplitude and phase of the motion, with a positional accuracy of 0.57º and highly stable frequency output.

Existing research suggests that the primary aerodynamic force inducing galloping acts perpendicular to the transmission line direction. Furthermore, ice accretion is typically assumed to be uniformly distributed along the conductor axis. Based on these premises, the aerodynamic parameter tests were justifiably simplified to a two-dimensional framework. A truncated sectional model was utilized for this purpose, with a height of 710 mm, as annotated in the schematic of Fig. 1c. To approximate two-dimensional flow conditions and minimize three-dimensional end effects, large, smooth circular end plates were installed at both ends of the conductor model. A general view of the experimental system is provided in Fig. 1c.

**Fig. 2:**
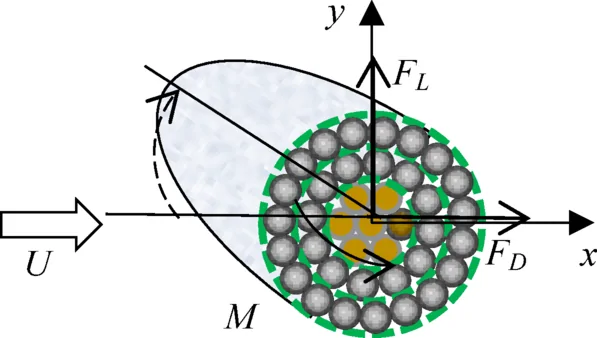
Cross section of crescent-shaped iced single conductor.

The aerodynamic forces acting on the conductor model under wind loading were measured via a force sensor mounted at the upper end of the model. An internal strain-gauge balance, housed within the model cavity, was used to simultaneously measure the three aerodynamic force components: lift, drag, and moment. The specific sensor employed was a model TG0151B. It features a measurement range of ± 60 N for both lift and drag forces, with a full-scale accuracy of 0.5%. The moment measurement range is ± 1 N·m, also with a full-scale accuracy of 0.5%. Additionally, the sampling frequency of the force sensor is set to 128 Hz, ensuring the timely and accurate collection of dynamic force data See Fig 2.

### Wind tunnel tests for aerodynamic coefficients

The aerodynamic coefficients of the crescent-shaped iced conductors include lift, drag and moment coefficients. The aerodynamic coefficients of the dimensionless crescent-shaped iced conductors can be defined, respectively, as the follows:

1 $${C_D}=\frac{{2{F_D}}}{{\rho {U^2}Ld}},\,{C_L}=\frac{{2{F_L}}}{{\rho {U^2}Ld}},\,{C_M}=\frac{{2M}}{{\rho {U^2}L{d^2}}}$$

Where *F*_*L*_, *F*_*D*_ and *M* are the lift, drag and torsional moment, respectively, *ρ* is the density of the air, *U* is the wind velocity, *L* is the length of the conductor, *d* is the diameter of the bare conductor.

First, the three aerodynamic loads acting on the steady iced conductors are obtained through wind tunnel tests, and then the corresponding three aerodynamic coefficients are calculated according to Eq. (1), and the aerodynamic coefficients can be used for investigation of galloping. This is the previous calculation method for aerodynamic parameters of iced conductors. In this paper, in order to investigate the influence of the movement of the iced conductors on aerodynamic parameters, the iced conductors in the test mode are rotated around the central axis at a certain pitching frequency, and then obtained the aerodynamic coefficients under unsteady response, the aerodynamic coefficients under unsteady conditions are also obtained by Eq. (1). The model’s rotation is controlled by a servo-motor system capable of both steady positioning and continuous unsteady oscillation. The test rig allows to perform “quasi-steady-state” tests. Observations of galloping of transmission lines show that, in most cases, the movement of iced conductors occurs within the range of wind velocities 4 m/s to 20 m/s. Therefore, during the wind tunnel test, the wind velocities were set to 10 m/s, 12 m/s, 14 m/s, and 18 m/s. The initial wind angle of attack is 0°, and the wind attack angle ranges from 0° to 360°, and the measurements of aerodynamic loads are performed every 5° to define the steady aerodynamic forces at different mean angles of attack. In the quasi-steady-state tests, data were sampled continuously for 5 s after the wind speed had been stabilized for 5 s, and the recorded data points were averaged over this sampling period. This approach effectively suppresses random errors induced by high-frequency fluctuations in the wind tunnel flow, small-amplitude vibrations of the model, and high-frequency electrical noise in the measurement system, thus yielding stable time-averaged static aerodynamic forces. “unsteady” tests providing a rotation with a cosine motion law at high frequency to investigate the unsteady aerodynamic coefficients of iced conductors, the pitching frequencies are 0.1 Hz, 0.2 Hz, 0.33 Hz, respectively. The initial wind attack angle is 0°, and the range of the wind attack angles from − 45° to 45°. For unsteady aerodynamic tests, aerodynamic parameters were sampled at an interval of 0.57°, and the data collected within each sampling interval were averaged.

It should be noted that a rigorous experimental uncertainty analysis was not conducted for the present wind tunnel tests. At high oscillation frequencies, the bin-averaging procedure (with a bin width of 0.57°) may lead to a reduced number of samples per bin, potentially impacting local data confidence. Notwithstanding these limitations, this study primarily aims to elucidate the variation patterns of aerodynamic parameters, thereby providing essential data support for numerical model calibration and neural network training. From the standpoints of trend analysis and model development, the experimental results remain highly relevant and possess practical significance.

## Wind tunnel test results

### Influence of pitching frequencies on unsteady aerodynamic coefficients

**Fig. 3:**
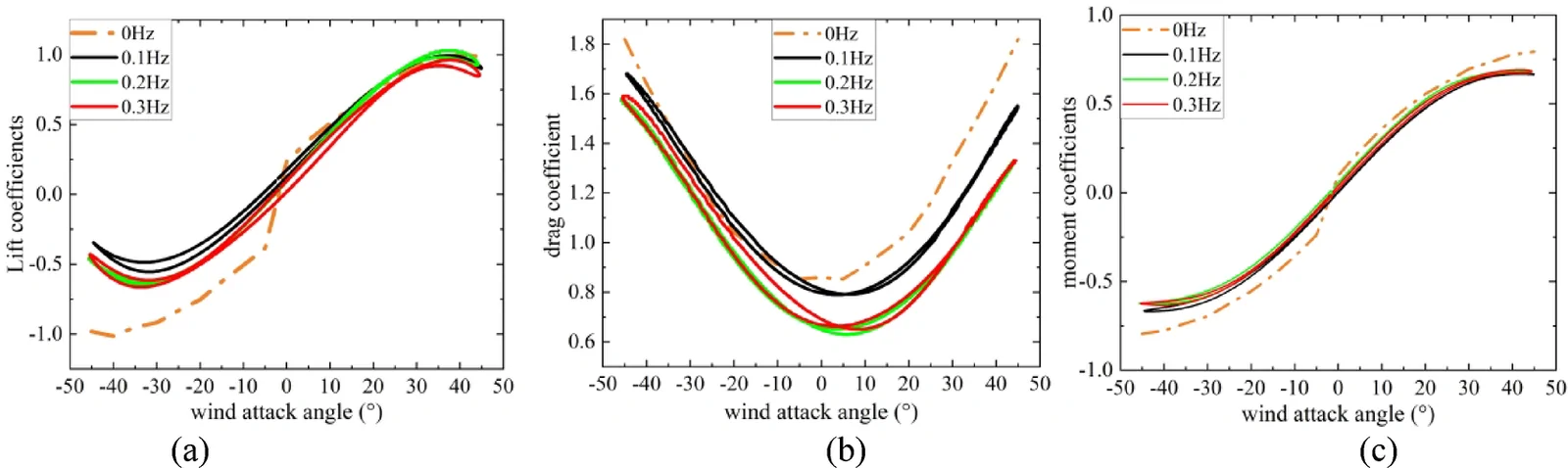
Comparison of aerodynamic coefficients of iced conductor varying with wind attack angle with different pitching frequencies under wind velocity of 10 m/s and ice thickness of 14 mm. (**a**) lift coefficients; (**b**) drag coefficients; (**c**) moment coefficients.

The aerodynamic coefficients of iced conductor varying with the wind attack angle under different pitching frequencies for conductors with ice thickness of 14 mm obtained by wind tunnel test are compared in Fig. 3, in which wind velocity is 10 m/s and wind attack angle is -45°~45°. Comparing the steady and unsteady aerodynamic lift coefficients of the iced single conductor as shown in Fig. 3a, it is seen that the curves of these two coefficients determined by the wind tunnel test have the same changing law are more consistent. However, unlike the steady aerodynamic lift coefficients curve, which only appears as a single line, the unsteady aerodynamic lift coefficients changes with the wind angle of attack as a closed loop. Because of airflow separation and vortex shedding at typical wind attack angle (Actual conductors in service operate in the trans-critical and supercritical Reynold’s number (Re) regime when in use on transmission lines. This means that vortex shedding frequencies are very high, and the wake structure is turbulent^23^.), the change of aerodynamic forces loaded on the conductor surface will lag behind the conductor’s motion state. As a result, the aerodynamic force shows a significant hysteresis characteristic with the change of the angle of attack. This phenomenon is named “aerodynamic hysteresis effect”.

Taking the aerodynamic coefficients in a vibration period as the ordinate and the dynamically changed attack angle as the abscissa, the curve of the aerodynamic coefficients changing with the wind attack angle—aerodynamic hysteresis curve can be obtained, which can not only indicate the relationship between the change of aerodynamic forces and the cross-sectional movement of the iced conductors, but also the area within the line can be used to evaluate the size of energy dissipated or input to the system by the aerodynamic forces, that is to describe the size and plus-minus of aerodynamic forces work in one cycle.

The area enclosed by the hysteresis loops is calculated using the Shoelace formula, also known as the Gauss area formula. This method computes the area of a simple polygon based on the coordinates of its vertices. For a polygon defined by a sequence of vertices P_1_(*x*_1_,*y*_1_), P_2_(*x*_2_,*y*_2_), …, P_*n*_(*x*_*n*_,*y*_*n*_), the area *A* is given by:

2 $$A=\frac{1}{2}\left| {\sum\limits_{{i=1}}^{n} {\left( {{x_i}{y_{i+1}} - {x_{i+1}}{y_i}} \right)} } \right|$$

where *x*_*n*+1_= *x*_1_ and *y*_*n*+1_= *y* to ensure closure of the polygon. The sign of the result depends on the vertex order: a clockwise sequence yields a positive value, while a counterclockwise sequence gives a negative value. In aerodynamic analysis, by treating the angle of attack *α* as the *x*-coordinate and the aerodynamic coefficient *C*_*i*_ (such as *C*_L_ or *C*_D_) as the *y*-coordinate, the area enclosed by the hysteresis loop can be quantitatively evaluated using this formula.

Among them, the positive or negative of aerodynamic forces work depends on the direction of the aerodynamic forces changing with the dynamic wind attack angle, and the size of the work depends on the area of the loop. The direction of rotation of the loop indicates the relative phase between force and displacement and therefore the energy sign: clockwise (+ circles) indicates energy pumping, anticlockwise (- circles) indicates energy dissipation^24,25^.

**Fig. 4:**
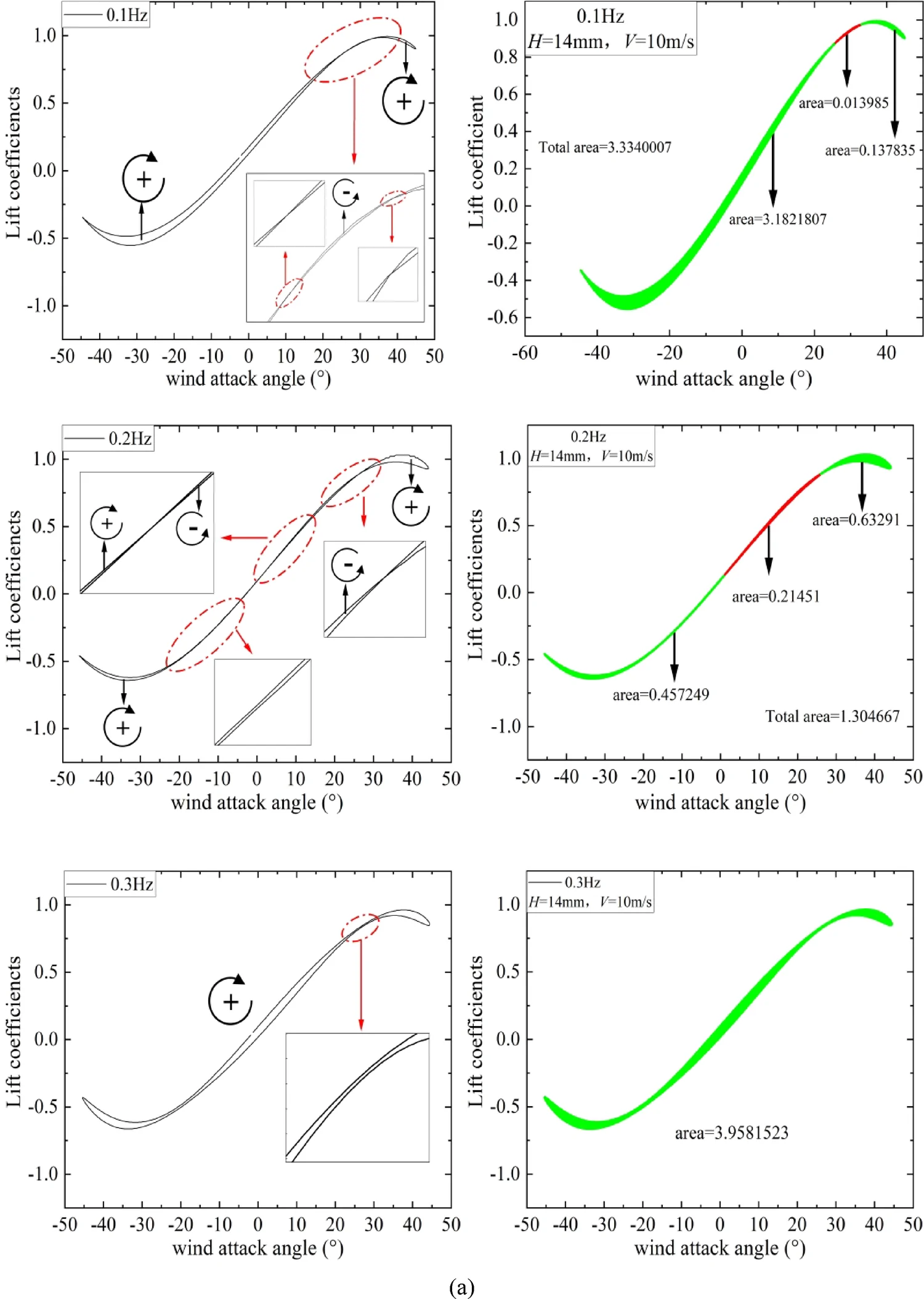

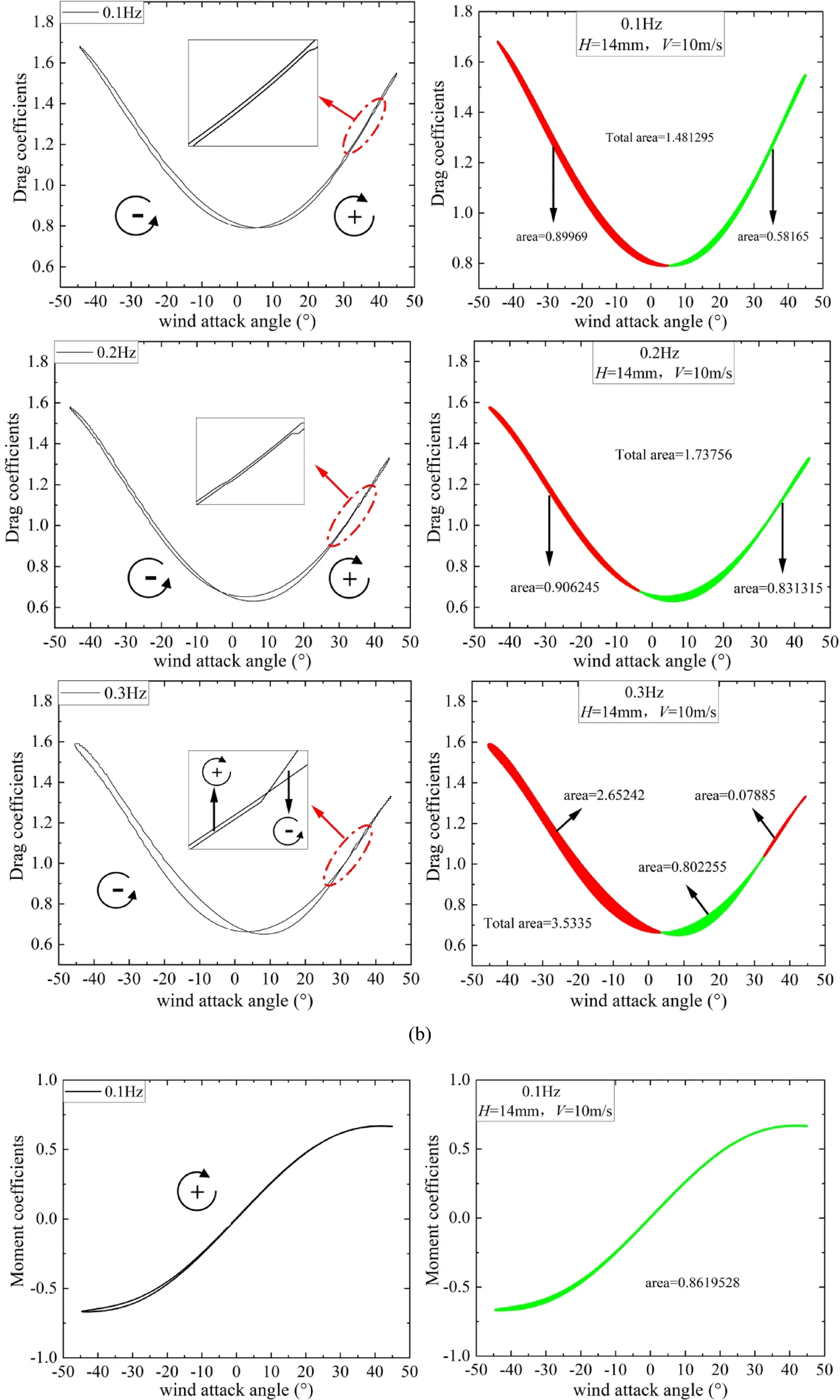

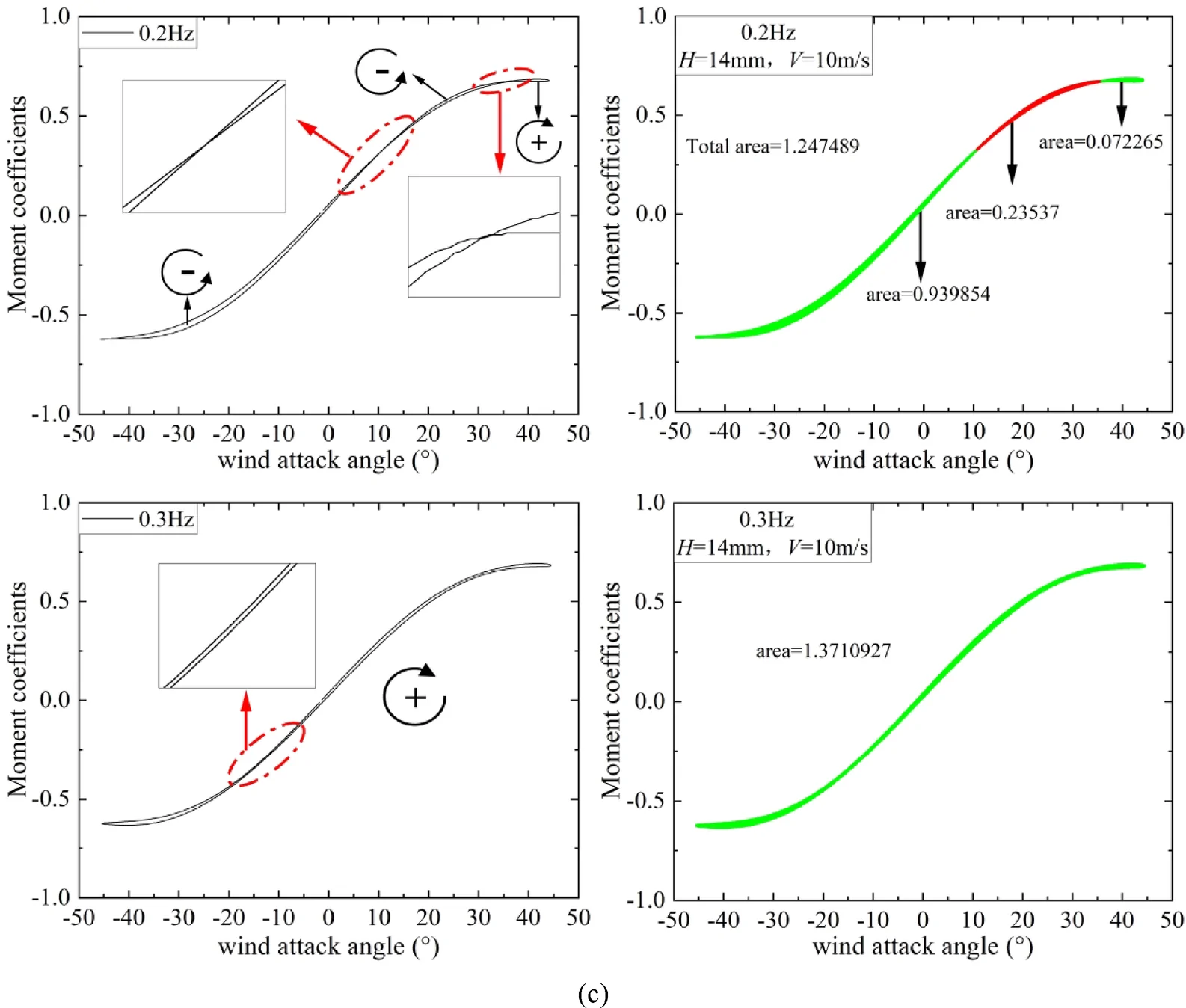
The hysteresis loops and the corresponding area of the aerodynamic coefficient under different pitching frequencies at wind velocity of 10 m/s and ice thickness of 14 mm. (**a**) lift coefficients; (**b**) drag coefficients; (**c**) moment coefficients.

At the same time, it can be seen from Fig. 3a that most of the steady aerodynamic lift coefficients are within the envelope range of the unsteady aerodynamic lift coefficients. At the beginning, the pitching frequency is 0.1 Hz, and the unsteady aerodynamic lift coefficients contain three loops (hysteresis loops), which indicates that the lift coefficients have both positive and negative work for the system at this time. By judging the direction of rotation of the three loops, it can be known that the left and right loops rotate clockwise—aerodynamic lifts do positive work, and the middle loop rotates counterclockwise—aerodynamic lifts do negative work. Then, comparing the area occupied by each loop, the area of the loop where the aerodynamic lift does the positive work is obviously larger than the area of the negative work part, they are 3.3200 and 0.0139 respectively (see Table 1), which means that the aerodynamic lift does positive work on the system. (In addition, in order to more intuitively and clearly show the number of hysteresis loops in each curve, the rotation direction and the area of each loop, Fig. 4 is given. Among them, the green filled part in the area diagram represents the area where the aerodynamic force does positive work, and the red filled part represents negative work). As the pitching frequency increases from 0.1 Hz to 0.2 Hz, the number of hysteresis loops remains three, and combines with Table 1; Fig. 3a it can be clearly seen that the area of the loop where the aerodynamic forces do positive work has been reduced, but it still takes up more of the loop area than the negative work part, indicating that the aerodynamic force still performs positive work on the system, and playing the role of energy input. When the pitching frequency continues to increase to 0.3 Hz, there is only one loop in the entire curve, and the direction of rotation remains clockwise, the aerodynamic forces also do positive work. And at this time, the area of the hysteresis loop is larger than the area of the hysteresis loop where the aerodynamic forces do positive work when the pitching frequencies are 0.1 Hz and 0.2 Hz, which shows that the aerodynamic lift do the maximum positive work to the system when the pitching frequency is 0.3 Hz (see Table 1). In addition, with the increase of the pitching frequencies, the lift coefficients all have different degrees of downward shift.

Similarly, part of the steady aerodynamic drag coefficients is within the envelope range of the unsteady aerodynamic drag coefficients, as shown in Fig. 3b. When the pitching frequency is 0.1 Hz, the unsteady aerodynamic drag coefficients curve contains two hysteresis loops, as a whole presenting a “8-shaped loop” with middle low and two sides high. This curve also shows that in a vibration period, the aerodynamic force does both positive and negative work, in which the left loop is the part where the aerodynamic force does negative work, and the right loop is the part of positive work. The drag coefficients curves under the same wind velocity, regardless of the value of the pitching frequency, the number of hysteresis loops contained in the curve is always two. As the pitching frequency increases from 0.1 Hz to 0.2 Hz, the drag coefficients have shown an overall downward shift, and the area of the left loop decreases (the part of negative work), and the area of the right loop increases (the part of positive work), but overall, the area of left loop is always greater than the area of the right loop. As the pitching frequency continues to increase to 0.3 Hz, the area of the drag coefficients curve increases, and the 0.2 Hz drag coefficients curve is nearly within its envelope range. The area of the negative work part of the aerodynamic force is significantly larger than the first two cases (0.1 Hz and 0.2 Hz), and the area of the right loop where the aerodynamic force does positive work is also larger. But, in general, the area of the left loop (the part of negative work) is larger than the area of the right loop (the part of positive work), which also indicates that the aerodynamic drag exerts negative work on the entire system and consumes energy.

Unlike the lift and drag hysteresis curves at various pitching frequencies, the moment coefficients curve does not show obvious offset phenomenon. On the whole, the curves show almost the same phenomenon as shown in Fig. 3c. However, through further analysis, it can be seen that the number of moment hysteresis loops will change as the pitching frequency increases. When the pitching frequency increases from 0.1 Hz to 0.2 Hz, the number of loops changes from the original one to three, it shows that part of the aerodynamic force produces positive damping and consumes energy at this time.

By judging the direction of rotation of the curve, the positive or negative of the aerodynamic force work represented by each loop can be determined: When the pitching frequency is 0.1 Hz, the curve rotates clockwise, and the aerodynamic force does positive work; when the pitching frequency is 0.2 Hz, the plus-minus of aerodynamic force work from left to right are: positive-negative-positive, respectively. And in the three loops, the area where the aerodynamic force does positive work is larger than the area of the negative work part. Therefore, regardless of whether the pitching frequencies are 0.1–0.2 Hz, the aerodynamic force plays the role of input energy to the motion system. If the pitching frequency continues to increase to 0.3 Hz, the number of loops becomes one again, and the aerodynamic force is still doing positive work.

It is worth noting that with the increase of the pitching frequency, the area of the aerodynamic force doing positive work in the lift coefficients curve first decreases and then increases, while the area of the aerodynamic force doing negative work in the drag coefficients curve and the area where the aerodynamic force does positive work in the moment coefficients curve keep increasing (only the area where the aerodynamic force does the main work is considered). In order to further analyze the law, the Tables 2 and 3 respectively gives the hysteresis loop area data of each aerodynamic coefficient under different pitching frequencies at the ice thickness of 24 mm and 33 mm. Combining the data given in the Table 2 and the Table 3 can be found, the area where the aerodynamic forces do positive work in the lift coefficients curves: (1) In the case of the ice thickness of 24 mm and 33 mm, it will increase with the increase of pitching frequency; (2) When the ice thickness is 14 mm, as the pitching frequency increases, the area first decreases and then increases. The area of aerodynamic force doing negative work in the drag coefficients curves: (1) In the case of the ice thickness of 14 mm and 33 mm, it will increase with the increase of pitching frequency; (2) When the ice thickness is 24 mm, with the increase of pitching frequency, the area first decreases and then increases. The area where the aerodynamic force does positive work in the moment coefficients curves: when the ice thicknesses are 14 mm, 24 mm, and 33 mm, they all increase as the pitching frequency increases. Based on the above results, it can be seen that the increase of pitching frequency will increase the work done by aerodynamic force in most cases, but the phenomenon that the area in the lift and drag coefficient curves first decreases and then increases at the closer ice thickness of 14 mm and 24 mm may be caused by measurement errors in the wind tunnel test. To elaborate, the primary source of measurement uncertainty is the TG0151B strain balance, which has a full-scale accuracy of 0.5% as stated in the methodology. For a measurement range of ± 60 N for lift and drag, this corresponds to an absolute uncertainty of approximately ± 0.3 N for both force components. However, some of the observed non-monotonic trends or slight variations in the hysteresis loop areas are of a magnitude comparable to this estimated measurement uncertainty band.

**Table 1:** The area of the hysteresis loops of the aerodynamic coefficients under different pitching frequencies at wind velocity of 10 m/s and ice thickness of 24 mm. (a) lift coefficients; (b) drag coefficients; (c) moment coefficients.

Pitching frequency	Number of loops	Area of positive work	Area of negative work	Total area
(a)				
0.1 Hz	3	3.32	0.01	3.33
0.2 Hz	3	1.09	0.21	1.30
0.3 Hz	1	3.96	0	3.96
(b)				
0.1 Hz	2	0.58	0.90	1.48
0.2 Hz	2	0.83	0.91	1.74
0.3 Hz	3	0.80	2.73	3.53
(c)				
0.1 Hz	1	0.86	0	0.86
0.2 Hz	3	1.01	0.24	1.25
0.3 Hz	1	1.37	0	1.37

**Table 2:** The area of the hysteresis loops of the aerodynamic coefficients under different pitching at wind velocity of 10 m/s and ice thickness of 24 mm. (a) lift coefficients; (b) drag coefficients; (c) moment coefficients.

Pitching frequency	Number of loops	Area of positive work	Area of negative work	Total area
(a)				
0.1 Hz	3	2.10	0.61	2.71
0.2 Hz	1	4.19	0	4.19
0.3 Hz	1	4.86	0	4.86
(b)				
0.1 Hz	3	0.10	2.41	2.51
0.2 Hz	2	1.55	1.88	3.43
0.3 Hz	2	0.58	2.29	2.87
(c)				
0.1 Hz	2	1.30	0.06	1.36
0.2 Hz	1	2.78	0	2.78
0.3 Hz	1	5.41	0	5.41

**Table 3:** The area of the hysteresis loops of the aerodynamic coefficients under different pitching at wind velocity of 10 m/s and ice thickness of 33 mm. (a) lift coefficients; (b) drag coefficients; (c) moment coefficients.

Pitching frequency	Number of loops	Area of positive work	Area of negative work	Total area
(a)				
0.1 Hz	2	9.51	0.24	9.75
0.2 Hz	3	15.76	0.41	16.17
0.3 Hz	3	22.26	0.66	22.92
(b)				
0.1 Hz	4	1.19	2.21	3.40
0.2 Hz	4	1.16	3.85	5.01
0.3 Hz	4	1.35	5.80	7.15
(c)				
0.1 Hz	3	9.54	0.06	9.60
0.2 Hz	1	14.69	0	14.69
0.3 Hz	3	20.87	0.09	20.96

### Influence of wind velocities on unsteady aerodynamic coefficients

**Fig. 5:**
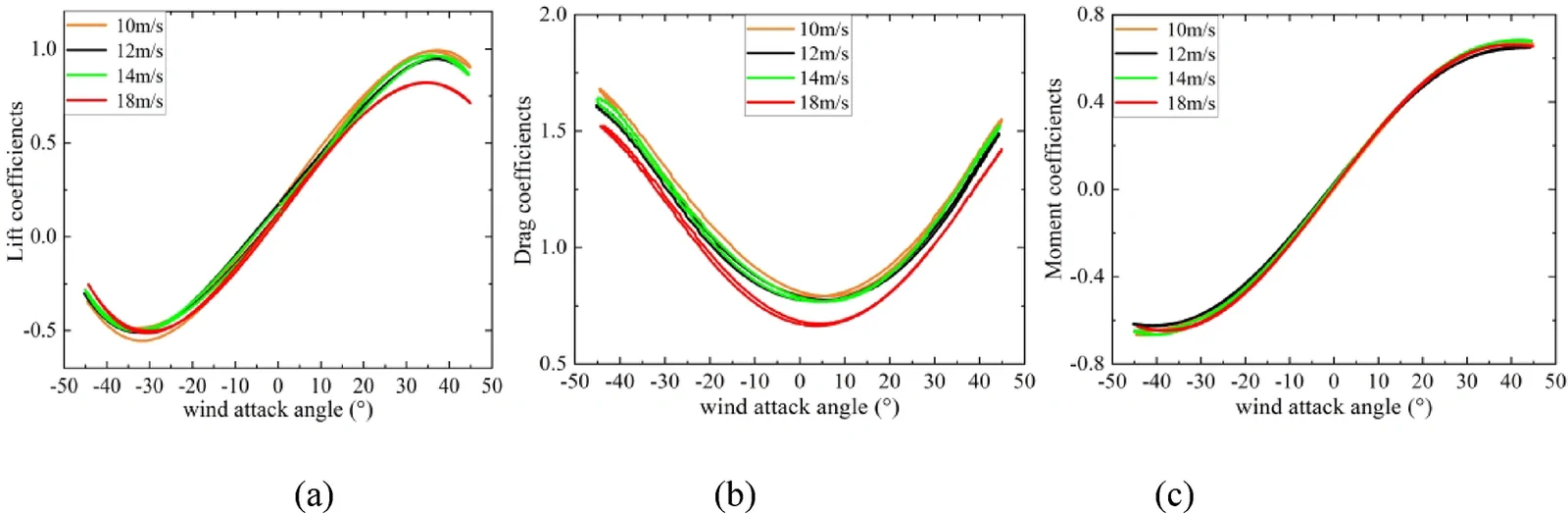
The aerodynamic coefficients of the iced conductor with 14 mm thickness ice and pitching frequency of 0.1 Hz under different wind velocities. (**a**) lift coefficients; (**b**) drag coefficients; (**c**) moment coefficients.

In order to research the influence of wind velocity on unsteady aerodynamic coefficients, the unsteady aerodynamic parameters of crescent-shape iced conductor under typical wind velocity (10 m/s, 12 m/s, 14 m/s, and 18 m/s) are measured. The unsteady aerodynamic lift coefficients of conductor with ice thickness of 14 mm varying with wind attack angle under different wind velocities are shown in Fig. 5a, in which pitching frequency is 0.1 Hz. The curve contains three hysteresis loops with the wind velocity of 10 m/s. By judging the direction of aerodynamic coefficients changing with the dynamic angle of attack, it can be seen that two of the three loops rotate clockwise -- doing positive work, and one rotates counterclockwise -- doing negative work. On the whole, the positive work done by aerodynamic force is greater than the negative work (see Table 4a). The number of hysteresis loops included in the lift coefficients curves will be affected by wind velocity. It can be seen from Table 4b and Table 4c that as the wind velocity increases to 12 m/s, the number of hysteresis loops is reduced to two, among which the positive work done by the aerodynamic force is far greater than the negative work. However, the number of hysteresis loops increases to three for the wind velocity reaches 14 m/s, and there is no longer a significant difference in the area of the loops where the aerodynamic force does positive work and negative work. If the wind velocity is further increased to 18 m/s, the number of loops remains at three, but the area of each loop becomes very small, close to a line, and closer to the state of the steady aerodynamic three-component force curve.

By comparing the three curves, it can be seen that the overall unsteady aerodynamic lift coefficients curve under different wind velocities is almost the same. Only when the wind velocity reaches 18 m/s and the angle of attack is greater than 20°, the curve has a significant change that the lift coefficients have a significant decrease. At the same time, as the wind velocity increases, the area of the entire loop gradually decreases, indicating that the work done by the aerodynamic lift on the system is getting smaller and smaller, and the curve collapses towards a single line, thus becoming morphologically closer to the single-valued nature of a steady aerodynamic coefficient curve. This is because when the wind velocity increases, the relative velocity of the cross-section movement of the ice conductor decreases, and the dependence of the change of aerodynamic force on the cross-section movement of the conductor reduces, thereby weakening the hysteresis effect, making the overall movement performance of the system gradually approach a steady state. In addition, the area of the loop where the aerodynamic lift does positive work decreases as the wind velocity increases.

The unsteady aerodynamic drag coefficients of conductor with ice of 14 mm thickness varying with wind attack angle under different wind velocities are shown in Fig. 5b, in which pitching frequency is 0.1 Hz. Be different from the lift coefficients curves, the number of hysteresis loops included in the drag coefficients curves not be affected by wind velocity, and always remain at two.

By judging the direction of rotation of the curves, it can be seen that the left loop represents the aerodynamic force does negative work, and the right loop represents the aerodynamic force does positive work. At this time, the area enclosed by the left loop is larger than the right loop, which indicates that the aerodynamic drag exerts negative work on the motion system and energy is dissipated, thereby reducing the amplitude of the pitching vibration of the conductor. As the wind velocity further increases, the gap between the negative and positive parts of the aerodynamic force becomes larger and larger (the part of negative > the part of positive). And the greater the wind velocity, the smaller the area of the loop on the right side, and even gradually approaching the line state, indicating that the aerodynamic drag always keeps doing negative work on the system. At the same time, as the wind velocity increases, the curve shows an overall downward trend, that is, the drag coefficients are continuously decreasing, but the area of the loop where aerodynamic drag does negative work increases first and then decreases.

The unsteady aerodynamic moment coefficients of conductor with ice of 14 mm thickness varying with wind attack angle under different wind velocities are shown in Fig. 5c, in which pitching frequency is 0.1 Hz. The curve has only one hysteresis loop for wind velocity is 10 m/s, and it rotates clockwise with the dynamic angle of attack, indicating that the aerodynamic force does positive work. If the wind velocity further increases (12 m/s, 14 m/s), the number of loops in the curve increases from one to three, where the left and right loops represent that the aerodynamic force does positive work, and the middle loop is the part of negative work. When the wind velocity reaches 14 m/s, among the three loops, only the left loop has a larger area, and the other two loops have a small area, indicating that the aerodynamic force still does positive work on the system at this time, and the pitching vibration amplitude of the conductor will keep increasing. When the wind velocity continues to increase to 18 m/s, the number of loops reduces to two, and the curve as a whole is very close to a line. As far as the overall phenomenon of the unsteady moment aerodynamic coefficients curves change with the wind velocity, there is basically no difference between the moment curves.

However, combining with the data in the Table 4, it can be seen that under the conditions of wind velocities of 12 m/s and 18 m/s, the area of negative work done by aerodynamic force is larger than that of positive work, indicating that the torsion amplitude of the conductor will decrease at this time. It can be inferred that when the wind velocity increases from 10 m/s to 12 m/s and 14 m/s to 18 m/s, the work done by aerodynamic force may pass through the moment when the positive and negative areas are equal, and the structure will be in a balance state of energy input and dissipation. But due to the lack of more detailed experimental data, it is temporarily unable to confirm the actual transformation process of the aerodynamic force does work.

**Table 4:** The area of the hysteresis loops of the aerodynamic coefficients under different wind velocities at pitching frequency of 0.1 Hz and ice thickness of 14 mm. (a) lift coefficients; (b) drag coefficients; (c) moment coefficients.

Wind velocity (m/s)	Number of loops	Area of positive work	Area of negative work	Total area
(a)				
10	3	3.32	0.01	3.33
12	2	1.68	0.08	1.75
14	3	1.37	0.85	1.37
18	4	0.73	0.21	0.94
(b)				
10	2	0.58	0.90	1.48
12	2	0.53	1.24	1.77
14	2	0.10	1.17	1.27
18	1	0	1.12	1.12
(c)				
10	1	0.86	0	0.86
12	3	0.10	0.22	0.32
14	3	0.40	0.23	0.63
18	2	0.23	0.24	0.47

### Influence of ice thickness on unsteady aerodynamic coefficients

**Fig. 6:**
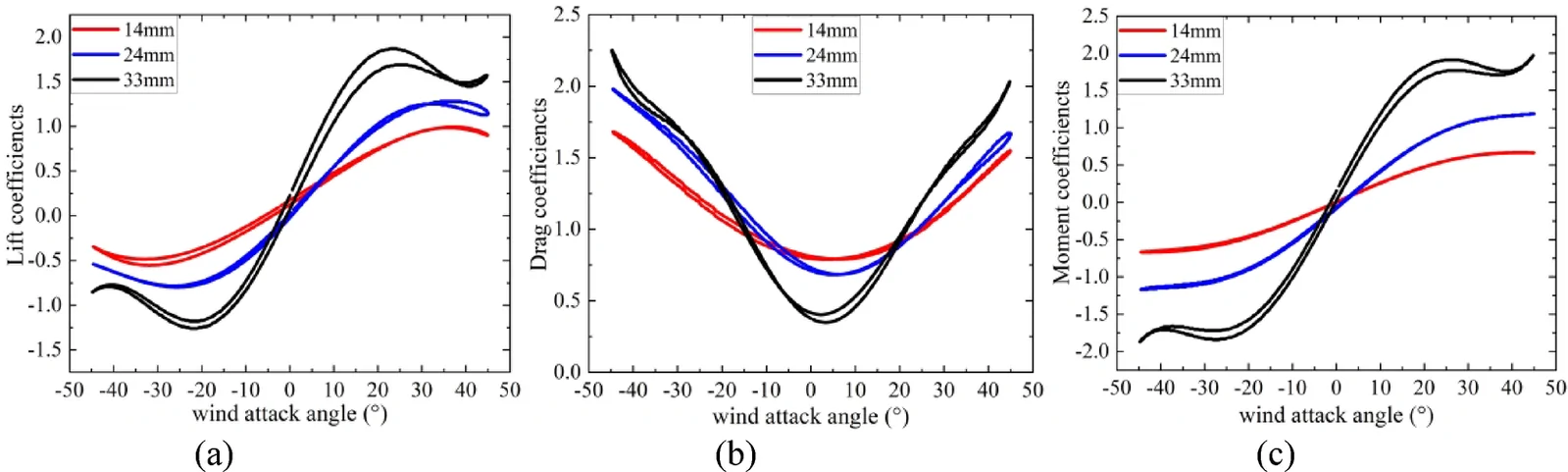
Comparison of aerodynamic coefficients of iced conductor varying with wind attack angle with different ice thickness under wind velocity of 10 m/s and pitching frequency of 0.1 Hz. (**a**) lift coefficients; (**b**) drag coefficients; (**c**) moment coefficients.

Furthermore, the ice thickness may also affect the aerodynamic characteristics of the iced conductors. The curves of the lift, the drag and the torsional moment coefficients of the iced conductors versus wind attack angle under different ice thickness are shown in Fig. 6. Figure 6a shows the unsteady lift coefficients curves with ice thickness of 14 mm, 24 mm, and 33 mm respectively. It can be seen intuitively that the lift coefficients curves of different ice thickness have obvious differences. Compared to the lift coefficients curve with the ice thickness of 14 mm, the lift coefficients curves for the ice thickness are 24 mm and 33 mm are roughly based on the 0° wind attack angle, and the left and right sides have downward and upward offsets respectively, and the greater the ice thickness, the greater the deviation, that is, the values of the lift coefficients increase with ice thickness, but the changing law of each coefficient is similar in the cases of different ice thickness. When the ice thickness is 14 mm, the curve contains three hysteresis loops, each of which represents the work done by the aerodynamic force (from left to right): positive-negative-positive. Because the area occupied by the negative work is small, the aerodynamic lift mainly does positive work on the system. The number of hysteresis loops in the curve is still three at the ice thickness of 24 mm, but at this time, the area of the middle loop that represents the aerodynamic force does negative work has increased significantly, and the area of the left and right loops has a certain amount decrease and increase respectively, but overall aerodynamic lift still does positive work. When the ice thickness reaches 33 mm, the number of loops of the lift coefficients curve is reduced to two, in which the loop on the right represents the aerodynamic lift does negative work, and the left loop represents the aerodynamic lift does positive work, and at this time the area of the curve is significantly larger than the other two cases, especially the loop area of the positive work part is the largest of the three, that means lift does more work. The positive work part is greater than the negative work part, indicating that the aerodynamic lift still does positive work to the system at this time, and the pitching amplitude of the conductor will continue to increase. At the same time, as the ice thickness increases, the slope of the curves increases, indicating that the nonlinearity increases.

Figure 6b shows the unsteady drag coefficients curves under different ice thickness. The minimum value of aerodynamic coefficient of drag curve decreases with the increase of the ice thickness, while the maximum values of aerodynamic coefficients on the left and right sides increase continuously. The overall performance is a funnel shape with a decreasing angle. Similarly, the changing law of each drag coefficient is similar in the cases of different ice thickness. The number of hysteresis loops included in the curve has increased from two with ice thickness of 14 mm to three with ice thickness of 24 mm and four with ice thickness of 33 mm. In the cases of the curve contains two hysteresis loops, the left loop represents the negative work, and the right loop represents the positive work, and the negative work part is obviously larger than the positive work part, and the pitching amplitude of the conductor will decrease. When the number of hysteresis loops increase to three, the left and right loops represent the negative work part, and the middle loop represents the positive work part, at this time the aerodynamic force also does negative work and the negative work part is larger than the case where the ice thickness is 14 mm. When the hysteresis loop increases to four, the area of the curve increases, and the aerodynamic force does work from left to right is: negative-positive-negative-positive, respectively. It can be found that the aerodynamic force still does negative work on the system.

Figure 6c shows the unsteady moment coefficients curves under different ice thickness (14 mm, 24 mm, 33 mm). The overall phenomenon shows a high similarity with the lift coefficients curves, that is, there are different degrees of deviation between each curve, but the changing law of each moment coefficient is still similar in the cases of different ice thickness. The number of hysteresis loops increase from the original one (14 mm) to two (24 mm), and as the ice thickness continues to increase, the number of loops also increase to three (33 mm). When there is only one loop, the direction of rotation of the curve is clockwise, indicating that the aerodynamic force does positive work at this time. When the number of loops becomes two, it means that part of the aerodynamic force starts to do negative work. By judgment, it can be known that the left loop represents positive work, and the right loop represents negative work. And it can be seen from the moment hysteresis curve with the ice thickness of 24 mm in the Fig. 13 and the data in Table 5 that the area of the left loop is larger than that of the right loop, so the aerodynamic force is also dominated by doing positive work. When the ice thickness increases to 33 mm, the area of the curve has increased significantly. The area of the loop on the left and right sides of the aerodynamic force does negative work is much smaller than the area of the loop doing positive work in the middle, indicating that the aerodynamic force still does positive work on the system and the energy in the system will increase.

Based on the above analysis of the influence of different pitching frequencies, wind velocities and ice thickness on the unsteady aerodynamic coefficients of the conductor, it can be seen that in terms of the numerical value of the aerodynamic coefficients, the pitching frequency has little influence on the lift and moment coefficients, but has a great influence on the drag coefficients, and this point also applies to the unsteady aerodynamic coefficients at different wind velocities. However, ice thickness has great influence on the three unsteady aerodynamic coefficients. In addition, in terms of work done by aerodynamic force: under pitching motion, the aerodynamic lift is always dominated by positive work, and the aerodynamic drag is dominated by negative work. But the moment coefficients, in the case of ice thickness of 14 mm and pitching frequency of 0.1 Hz, appear an overall conversion in aerodynamic work with the change of wind velocity. Besides, the aerodynamic moment is still dominated by positive work. In other words, under pitching conditions, the work done by lift and torque will cause the amplitude of the conductor to increase, while aerodynamic drag will reduce the amplitude of the conductor.

**Table 5:** The area of the hysteresis loops of the aerodynamic coefficients under different ice thicknesses at wind velocity of 10 m/s and pitching frequency of 0.1 Hz. (a) lift coefficients; (b) drag coefficients; (c) moment coefficients.

Ice thickness (mm)	Number of loops	Area of positive work	Area of negative work	Total area
(a)				
14	3	3.32	0.01	3.33
24	3	2.10	0.61	2.71
33	2	9.51	0.24	9.75
(b)				
14	2	0.58	0.90	1.48
24	3	0.10	2.41	2.51
33	4	1.19	2.21	3.40
(c)				
14	1	0.86	0	0.86
24	2	1.30	0.06	1.36
33	3	9.54	0.06	9.60

##  Unsteady aerodynamic coefficients based on quasi-static assumptions (MATLAB)

### The influence of wind velocities on unsteady aerodynamic coefficients based on quasi-static assumptions

**Fig. 7:**
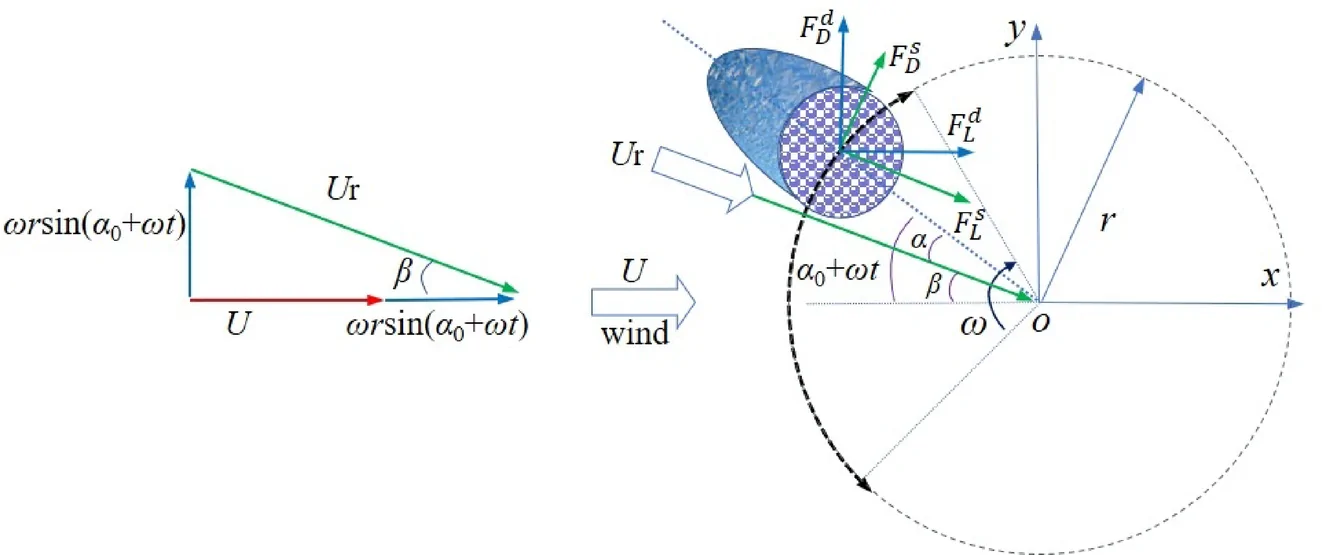
Schematic diagram for obtaining unsteady aerodynamic parameters.

The calculation model for obtaining unsteady aerodynamic parameters based on static aerodynamic parameters from wind tunnel tests is shown in Fig. 7. Among them, Fig. 7 can be regarded as a top view that combines Fig. 1 with Fig. 1c. Point O serves as the rotation center, and the iced conductor rotates around point O at a certain frequency. The distance from the conductor to the center of rotation is *r*.

*U* is the incoming wind velocity on the conductor. The vertical and horizontal velocities generated by the conductor’s motion are $\omega r\sin ({\alpha _0}+\theta )$and$\omega r\cos ({\alpha _0}+\theta )$,respectively. *θ* is the angle through which the iced conductor rotates around the axis O, and *α*_0_ is the initial angle of the iced conductor. Under the superimposed velocities in both vertical and horizontal directions, the relative wind velocity, *U*r, and the change in angle of attack, *β*, are generated. According to the Pythagorean theorem, the relative wind velocity shown in the figure can be expressed as:

3 $${U_{\mathrm{r}}}=\sqrt {{{[\dot {\theta }r\cos ({\alpha _0}+\theta )]}^2}+{{[U+\dot {\theta }r\sin ({\alpha _0}+\theta )]}^2}}$$

as shown in the figure, the change in angle of attack for iced conductor can be expressed as:

4 $$\beta =\arctan [\frac{{\dot {\theta }r\cos ({\alpha _0}+\theta )}}{{U+\dot {\theta }r\sin ({\alpha _0}+\theta )}}]$$

Combining Eq. (4) with the angle of attack definition provided in Fig. 2, the angle of attack for the conductor’s motion can be obtained as:

5 $$\alpha ={\alpha _0}+\theta - \beta$$

With the determined relative wind velocity and relative angle of attack, the unsteady aerodynamic three-component forces acting on iced conductor can be calculated. This calculation relies on the quasi-steady assumption and leverages static aerodynamic parameters derived from wind tunnel experiments. Consequently, under the quasi-steady assumption, the aerodynamic lift, drag, and torque can be formulated as:

6 $$\begin{gathered} F_{L}^{S}=\frac{1}{2}\rho d{U_r}^{2}C_{L}^{S} \hfill \\ F_{D}^{S}=\frac{1}{2}\rho d{U_r}^{2}C_{D}^{S} \hfill \\ M_{{}}^{S}=\frac{1}{2}\rho d{U_r}^{2}C_{M}^{S} \hfill \\ \end{gathered}$$

where $F_{L}^{S}$,$F_{D}^{S}$*and* $M_{{}}^{S}$ are the static aerodynamic lift, drag, and torque, respectively, for the iced conductor. $C_{L}^{S}$, $C_{D}^{S}$ and$C_{M}^{S}$denote the static aerodynamic lift, drag, and torque coefficients, respectively, for the iced conductor. *ρ* is the local air density, and d is the bare conductor diameter.

As illustrated in Fig. 7, the static aerodynamic drag acts along the direction of the relative wind velocity, while the static aerodynamic lift is perpendicular to it. Consequently, by projecting these aerodynamic forces onto the vertical and horizontal directions, the unsteady aerodynamic lift and unsteady aerodynamic drag are obtained. Combining this with the aerodynamic force model derived previously, the unsteady aerodynamic lift, drag, and torque under the quasi-steady assumption can be expressed as:

7 $$\begin{gathered} F_{L}^{D}=F_{L}^{S}\cos (\beta ) - F_{D}^{S}\sin (\beta )=\frac{{\mathrm{1}}}{{\mathrm{2}}}\rho dU_{r}^{2}\left[ {C_{L}^{S}\cos (\beta ) - C_{D}^{S}\sin (\beta )} \right] \hfill \\ F_{D}^{D}=F_{L}^{S}\cos (\beta )+F_{D}^{S}\sin (\beta )=\frac{{\mathrm{1}}}{{\mathrm{2}}}\rho dU_{r}^{2}\left[ {C_{L}^{S}\cos (\beta )+C_{D}^{S}\sin (\beta )} \right] \hfill \\ M_{{}}^{D}=\frac{{\mathrm{1}}}{{\mathrm{2}}}\rho d{U_r}^{2}C_{M}^{S} \hfill \\ \end{gathered}$$

where $F_{L}^{D}$, $F_{D}^{D}$and $M_{{}}^{D}$denote the unsteady aerodynamic lift, drag, and torque, respectively, for the iced conductor, derived based on the quasi-steady assumption.

Based on Eq. (7), the unsteady aerodynamic coefficients for the iced conductor can be further derived as:

8 $$\begin{gathered} C_{L}^{D}=\frac{{2F_{L}^{D}}}{{\rho dU_{{}}^{2}}}={\left( {\frac{{{U_r}}}{U}} \right)^2}\left[ {C_{L}^{S}\cos (\beta ) - C_{D}^{S}\sin (\beta )} \right] \hfill \\ C_{D}^{D}=\frac{{2F_{D}^{D}}}{{\rho dU_{{}}^{2}}}={\left( {\frac{{{U_r}}}{U}} \right)^2}\left[ {C_{L}^{S}\cos (\beta )+C_{D}^{S}\sin (\beta )} \right] \hfill \\ C_{M}^{D}=\frac{{2M_{{}}^{{Dyn}}}}{{\rho d{U_{}}^{2}}}={\left( {\frac{{{U_r}}}{U}} \right)^2}C_{M}^{S} \hfill \\ \end{gathered}$$

where $C_{L}^{D}$, $C_{D}^{D}$ and $C_{M}^{D}$ represent the unsteady aerodynamic lift, drag, and torque coefficients, respectively, for the iced conductor, derived based on the quasi-steady assumption.

**Fig. 8:**
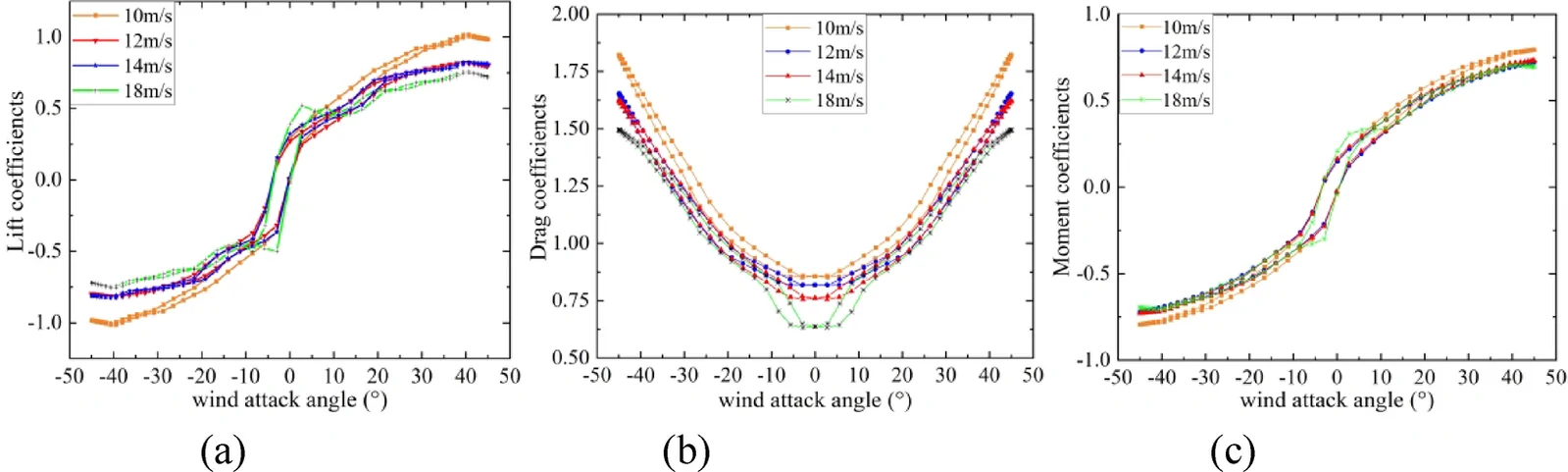
The unsteady aerodynamic coefficients of the iced conductor based on quasi-static assumption with 14 mm thickness ice and pitching frequency of 0.1 Hz under different wind velocities. (**a**) lift coefficients; (**b**) drag coefficients; (**c**) moment coefficients.

The unsteady aerodynamic coefficients under different wind velocities based on quasi-static assumptions obtained by MATLAB, as shown in Fig. 8. The changing laws of lift, drag and moment coefficients are similar, under different wind velocities. As shown in Fig. 8a, different from the unsteady lift aerodynamic coefficients obtained by wind tunnel test (under the same basic parameters), two of the three hysteresis loops included in this curve rotate counterclockwise: the aerodynamic force does negative work, and only one rotates clockwise: the aerodynamic force does positive work, but because of the area of the loop where the aerodynamic force does negative work is small. Therefore, the aerodynamic force is still based on doing positive work, which is consistent with the results of the wind tunnel tests. As the wind velocity increases to 12 m/s, the number of hysteresis loops and the direction of rotation remain unchanged, and the area of the middle loop is basically same in the range of -5.7°—2.7°, while the left and right sides of the curve have different degrees of downward and upward shift, respectively. When the wind velocity continues to increase to 14 m/s, the curve begins to envelop each other, and the aerodynamic force still does positive work on the system. If the wind velocity continues to increase to 18 m/s, the left and right sides of the curve once again have different degrees of downward and upward shift, and the number of loops increases from three to seven. Among them, three loops represent the aerodynamic forces do positive work, and the rest of the loops represent negative work. It can be seen from Table 6 that the area of the positive work part is obviously larger than the negative work part, indicating that the aerodynamic lift always does positive work on the system, and the pitching amplitude of the conductor continues to increase. At the same time, from the area data listed in Table 6, it can be seen that the area of the loop where the aerodynamic lift does positive work first decreases, then increases, and finally decreases with the increase of wind velocity. However, the increased value is very small, which may be caused by modeling errors or calculation errors. The overall trend is still decreasing. This rule is also consistent with the results of the wind tunnel test.

It can be seen from Fig. 8b that the number of hysteresis loops in the drag coefficients curves is gradually reduced from the initial four to two. When there are four hysteresis loops (10, 12 m/s), the plus-minus of work done by aerodynamic drag from left to right are, respectively: positive-negative-positive-negative. As the wind velocity increases to 14 m/s, 18 m/s, the number of hysteresis loops becomes two, in which the left loop represents aerodynamic drag does negative work, and the right loop represents positive work. At the same time, as the wind velocity increases, the aerodynamic coefficients appear to move downward overall. The local slope of the curve increases with the wind velocity of 18 m/s, which shows that the increase of wind velocity leads to the increase of system nonlinearity. In addition, it can be seen from Table 6 that the positive work and negative work done by the aerodynamic force are basically the same, indicating that the system will be in the balance stage of energy input and dissipation, and the conductor will keep a stable galloping amplitude. However, compared with the analysis of the aerodynamic coefficients of dynamic drag obtained by the wind tunnel test in the previous chapter, it can be seen that the aerodynamic drag should do negative work to the system. The difference of the work done to the system by the aerodynamic drag shown by the two methods may be related to the fact that the influence of relative wind velocity is not considered in the numerical model.

It can be seen from Fig. 8c; Table 6 that the unsteady moment aerodynamic coefficients curves at wind velocities of 10 m/s, 12 m/s and 14 m/s based on quasi-static assumptions all have only one hysteresis loop. The loop rotates clockwise, and at this time, the pitching amplitude of the conductor will increase due to the positive work done by the aerodynamic force. The continuous increasing of wind velocity has the same influence on the moment aerodynamic coefficients as the lift aerodynamic coefficients, and the same remains unchanged within the range of wind attack angle of 0°, but there are varying degrees of downward and upward shifts on the left and right sides. The number of hysteresis loops increase to three at the wind velocity of 18 m/s, and the aerodynamic force is still dominated by positive work, which is consistent with the results of the wind tunnel test. In addition, it can be seen from Table 6 that the area of loop represents the positive work in the moment coefficient curve decreases first, then increases, and finally decreases as the wind velocity increases. This rule is also consistent with the results of the wind tunnel test.

**Table 6:** The area of the hysteresis loops of the aerodynamic coefficients under different wind velocities at pitching frequency of 0.1 Hz and ice thickness of 14 mm (MATLAB). (a) lift coefficients; (b) drag coefficients; (c) moment coefficients.

Wind velocity (m/s)	Number of loops	Area of positive work	Area of negative work	Total area
(a)				
10	3	6.79	0.04	6.83
12	3	5.63	0.03	5.66
14	3	5.67	0.02	5.69
18	7	5.44	0.24	5.69
(b)				
10	4	2.21	2.21	4.42
12	4	1.87	1.87	3.74
14	2	2.08	2.05	4.12
18	2	2.31	2.30	4.61
(c)				
10	1	5.17	0	5.17
12	1	4.68	0	4.68
14	1	4.76	0	4.76
18	3	4.73	0.01	4.74

**Fig. 9:**
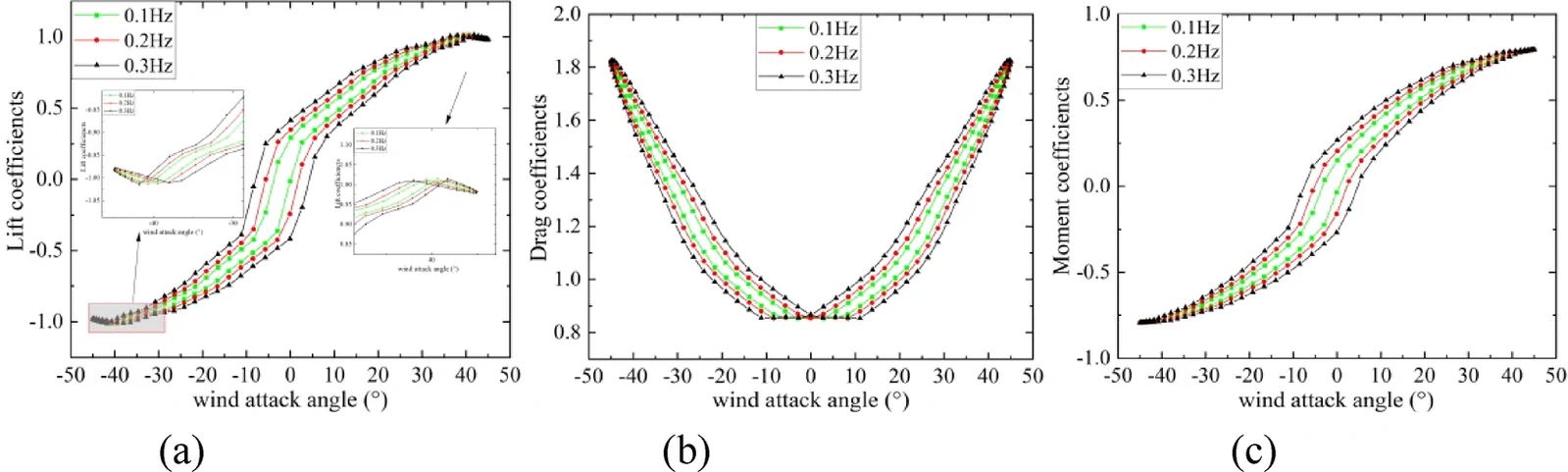
The unsteady aerodynamic coefficients of the iced conductor based on quasi-static assumption with 14 mm thickness ice and wind velocity of 10 m/s under different pitching frequencies. (**a**) lift coefficients; (**b**) drag coefficients; (**c**) moment coefficients.

As the torsion frequency increases, the envelope range of the unsteady aerodynamic coefficients curves gradually increases, and all of them show the phenomenon of large loops covering small loops, in which the increase in the area of the curve indicates the increase in doing work by aerodynamic force, as shown in Fig. 9. Comparing the unsteady aerodynamic coefficients obtained from wind tunnel test at different torsion frequencies shown in Fig. 9, it can be found that although the results of wind tunnel test also have a curve envelope phenomenon, this phenomenon is less and the regularity is not strong. At the same time, the curves also have a certain degree of deviation, and the number of hysteresis loops in the lift and moment coefficients curves has changed as the torsion frequency increases. However, in the results based on the quasi-static assumption, the number of hysteresis loops and the rotation direction of each loop remain unchanged.

The lift curve contains three hysteresis loops, but the aerodynamic lift does work is still dominated by positive work because of the area of the loops on the left and right sides that represent the aerodynamic force doing negative work is small. In addition, the lift coefficients curve is between the attack angle of -8°~-3° and − 3°~5°, and its curvature is large, which means the increase of nonlinearity. The phenomenon of the moment coefficients curves is similar to lift coefficients curves, but the number of hysteresis loops is only one and rotates clockwise, the aerodynamic force also does positive work on the system. For the drag coefficients curves, the number of hysteresis loops is two, the left loop represents the aerodynamic force does negative work, and the right loop represents the aerodynamic force does positive work. The area of the two loops in the drag coefficients curves is basically the same. The reason may also be that the numerical model does not consider the influence of the relative wind velocity on the aerodynamic coefficients. In addition, the area of the main work part done by the aerodynamic force in the lift, drag, and moment coefficients curves increases with the increase of the pitching frequency. Such as the positive work part in the lift and moment coefficients curves, the negative work part in the drag coefficients curves, this law is consistent with the results of the wind tunnel tests.

### The influence of ice thickness on unsteady aerodynamic coefficients based on quasi-static assumption

**Fig. 10:**
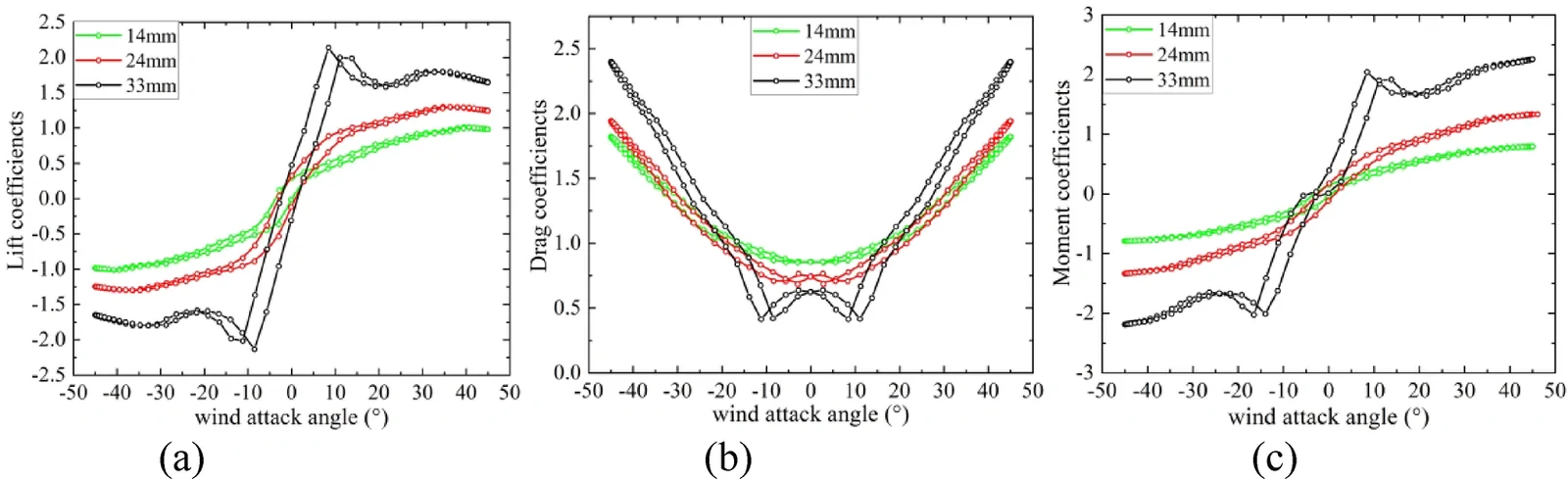
Comparison of aerodynamic coefficients of iced conductor based on quasi-static assumption varying with wind attack angle with different ice thickness under wind velocity of 10 m/s and pitching frequency of 0.1 Hz. (**a**) lift coefficients; (**b**) drag coefficients; (**c**) moment coefficients.

In order to analyze the influence of ice thickness on the unsteady aerodynamic coefficients based on the quasi-static assumption, the Fig. 10 shows the comparison of unsteady aerodynamic coefficients under different ice thickness. The lift, drag and moment coefficient curves all have different degrees of deviation, especially the deviation of the lift and moment coefficient curves is the most obvious. At the same time, the results of wind tunnel tests under different ice thickness have similar phenomena. Moreover, when the ice thickness reaches 33 mm, the slopes of the three curves all have a significant increase, which is also consistent with the results of the wind tunnel test, indicating that the ice thickness has a greater influence on the nonlinearity of the system.

The comparison of lift coefficients under different ice thickness is shown in Fig. 10a. When the ice thickness is 14 mm, there are three hysteresis loops. The left and right loops represent the negative work part, and the middle loop represents the positive work part. At this time, the positive work part is obviously larger than the negative work part. When the ice thickness increases to 24 mm, the number of hysteresis loops and the rotation direction of each loop remain unchanged, only the area of curve increases, especially the area of the positive work part increases significantly. If the ice thickness continues to increase to 33 mm, the number of hysteresis loops increases to seven, and the area of the curve increases again. Each loop represents the plus or minus of work done by aerodynamic force from left to right are: negative-positive-negative-positive-negative-positive-negative, respectively. Among them, the area where the aerodynamic force does positive work is still the largest. Based on the above conclusions, the phenomenon is basically consistent with the results of the wind tunnel test, and no matter how the ice thickness changes, the aerodynamic force does work is always dominated by positive work. At the same time, the area of positive work also increases with the increase of the ice thickness.

The comparison of drag coefficients under different ice thickness is shown in Fig. 10b, and the drag coefficients curve presents a symmetrical parabolic loop from − 45° to 45° with a middle low and two sides high. The number of hysteresis loops does not change with the change of ice thickness, it is always four. However, because of the drag coefficient curve is symmetrical with the 0° wind attack angle as the baseline, so no matter how many hysteresis loops are included in the curve, the area of the positive and negative work part is always the same. And the area of negative work also increases with the increase of the ice thickness. These phenomena are consistent with the phenomenon presented by the drag coefficients curves in Sect.  4.1 (Fig. 8b) and 4.2 (Fig. 9b) above, and the reason is also consistent with the above analysis.

As shown in Fig. 10c are the moment coefficients curves under different ice thickness. The number of hysteresis loops increases from one (14 mm, 24 mm) to five (33 mm), and the area also increases gradually. When there is only one hysteresis loop in the curve, the rotation direction of the loop is clockwise, and the aerodynamic force does positive work. The ice thickness increases from 14 mm to 24 mm, which increases the area of the loop, indicating that the aerodynamic force does more work. When the ice thickness reaches 33 mm, the number of hysteresis loops increases to as many as five. Each loop represents the plus or minus of work done by aerodynamic force from left to right are: positive-negative-positive-negative-positive, respectively, and the positive work part is greater than the negative work part. In addition, the local slope of the curve increases with the increase of ice thickness, especially at 33 mm, which indicates that the ice thickness can enhance the nonlinearity of the system. By comparing the above results with the moment hysteresis curves of wind tunnel test, it can be found that the laws of the curves in the two cases are the same, and the work done by the aerodynamic force is mainly positive work. The area of positive work also increases with the increase of the ice thickness.

Figure 10 shows that the ice thickness significantly influences the trend of hysteresis loops in the lift, drag, and moment coefficients. The hysteresis trends of the aerodynamic coefficients are relatively similar at ice thicknesses of 14 mm and 24 mm. However, when the ice thickness reaches 33 mm, localized segments of the hysteresis loops exhibit noticeable differences.

**Table 7:** The area of the hysteresis loops of the aerodynamic coefficients under different ice thicknesses at wind velocity of 10 m/s and pitching frequency of 0.1 Hz (MATLAB). (a) lift coefficients; (b) drag coefficients; (c) moment coefficients.

Ice thickness (mm)	Number of loops	Area of positive work	Area of negative work	Total area
(a)				
14	3	6.79	0.04	6.83
24	3	9.13	0.11	9.23
33	7	15.96	2.88	18.84
(b)				
14	4	2.22	2.22	4.44
24	4	3.10	3.10	6.20
33	4	5.20	5.21	10.40
(c)				
14	1	5.17	0	5.17
24	1	8.61	0	8.61
33	5	16.24	1.60	17.83

## Comparison of numerical and test results

### Comparison between unsteady aerodynamic coefficients based on quasi-static hypothesis and steady aerodynamic coefficients of wind tunnel tests

**Fig. 11:**
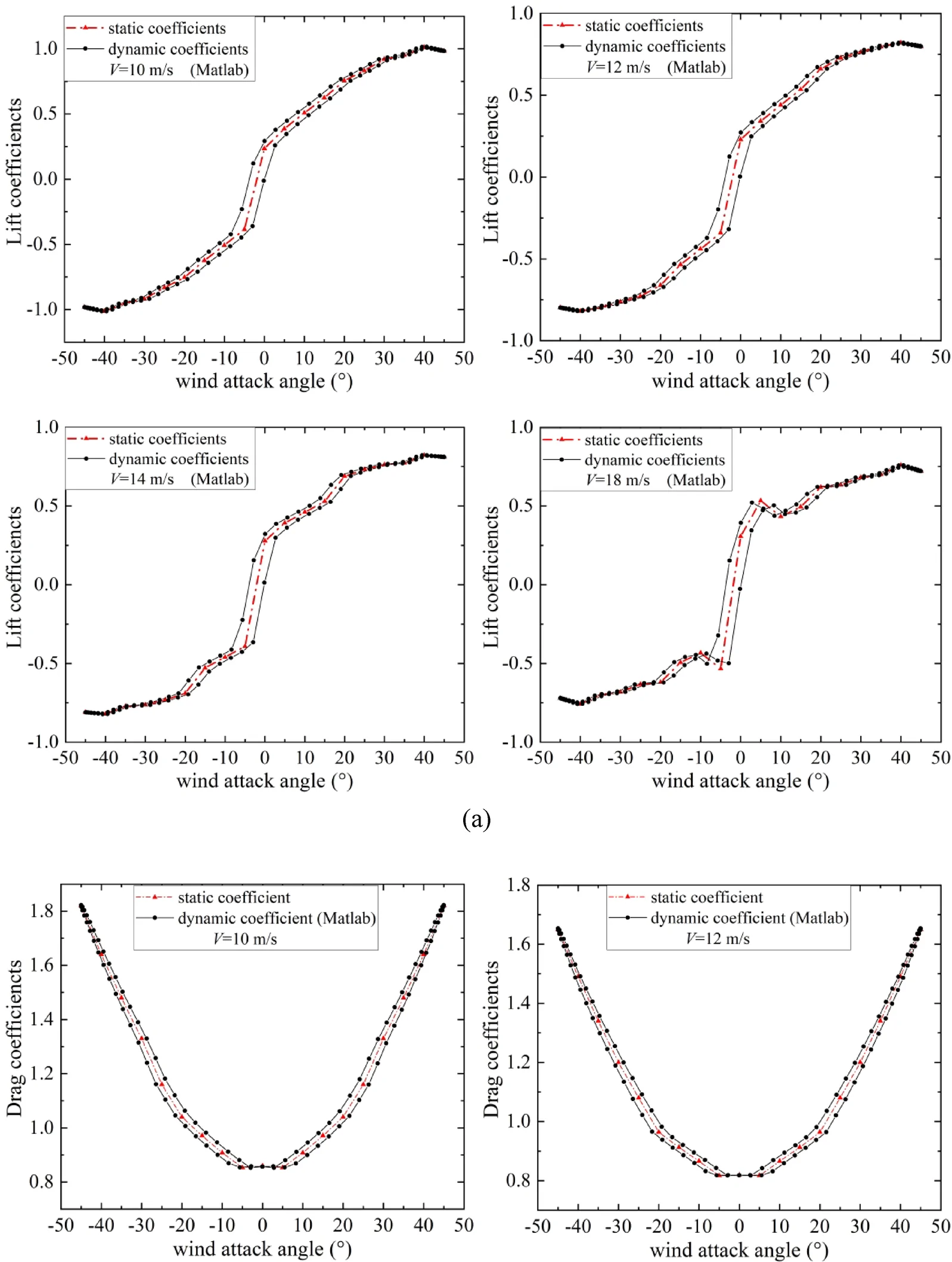

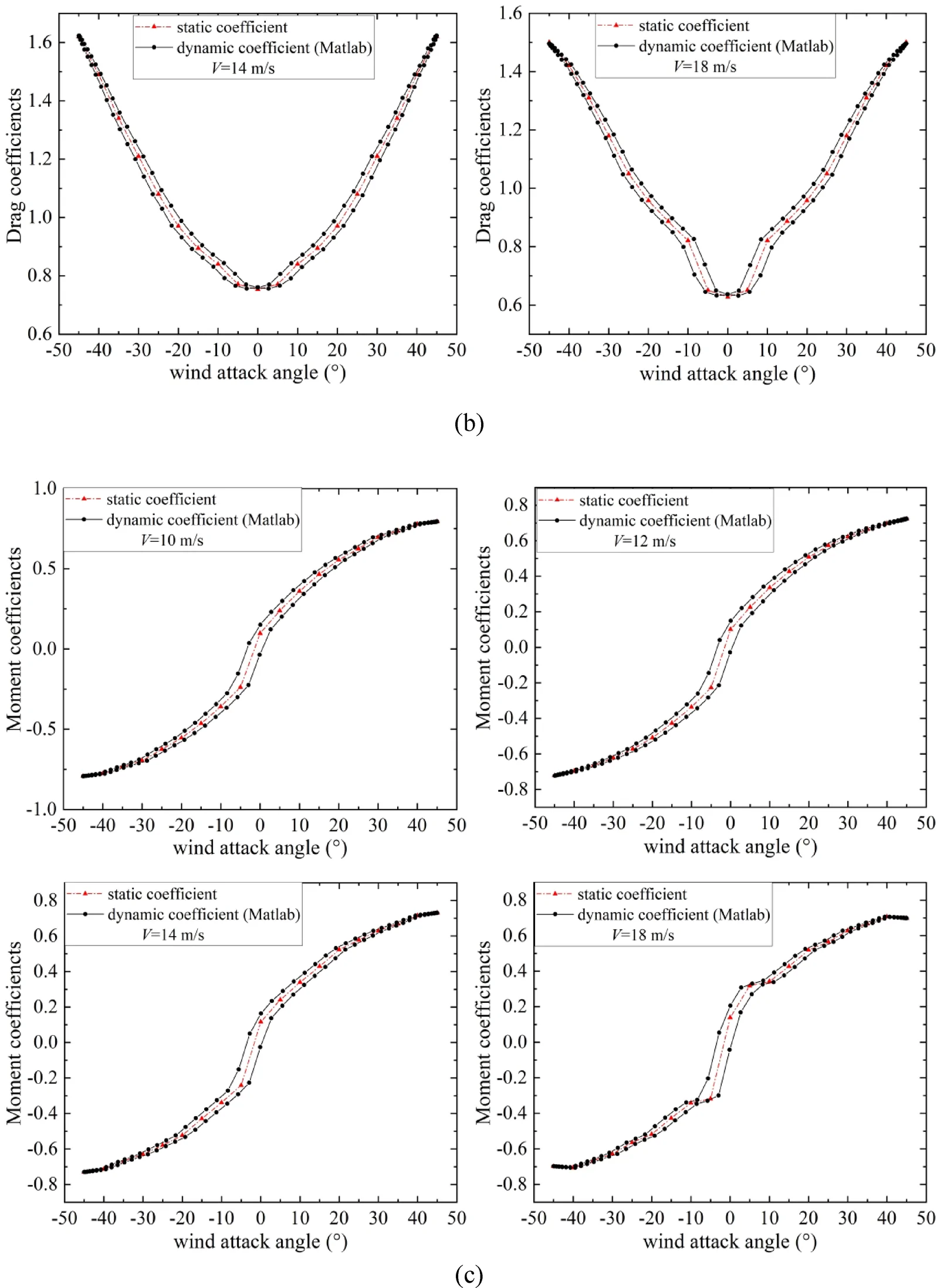
Comparison of unsteady aerodynamic coefficients based on quasi-static assumption and steady aerodynamic coefficients from wind tunnel test under different wind velocity with 14 mm thickness ice. (**a**) lift coefficients; (**b**) drag coefficients; (**c**) moment coefficients.

On the basis of the above analysis, then the unsteady aerodynamic coefficients curves based on quasi-static theory obtained by MATLAB and the steady aerodynamic coefficients curves obtained by wind tunnel test under different wind velocities are compared in this paper, as shown in Fig. 11. No matter how the wind velocity changes, each aerodynamic coefficients curve is always symmetrically distributed around the steady aerodynamic coefficients curve, presenting an envelope state, that is, the steady aerodynamic coefficient curve represents the average value of the hysteresis loop. In fact, the steady aerodynamic coefficients are obtained under the condition that the aerodynamic force is synchronized with the angle of attack. During the test, the instantaneous angle of attack will not be generated. But the unsteady aerodynamic coefficients are obtained at a certain pitching frequency, there will be a phase difference between the aerodynamic force and the instantaneous angle of attack, resulting in the curve being displayed as a loop, and if the force and the instantaneous angle of attack are completely in phase, the hysteresis loop will be displayed as a simple line, that is, the steady coefficient curve.

### Comparison of unsteady aerodynamic coefficients based on quasi-static hypothesis and wind tunnel test

**Fig. 12:**
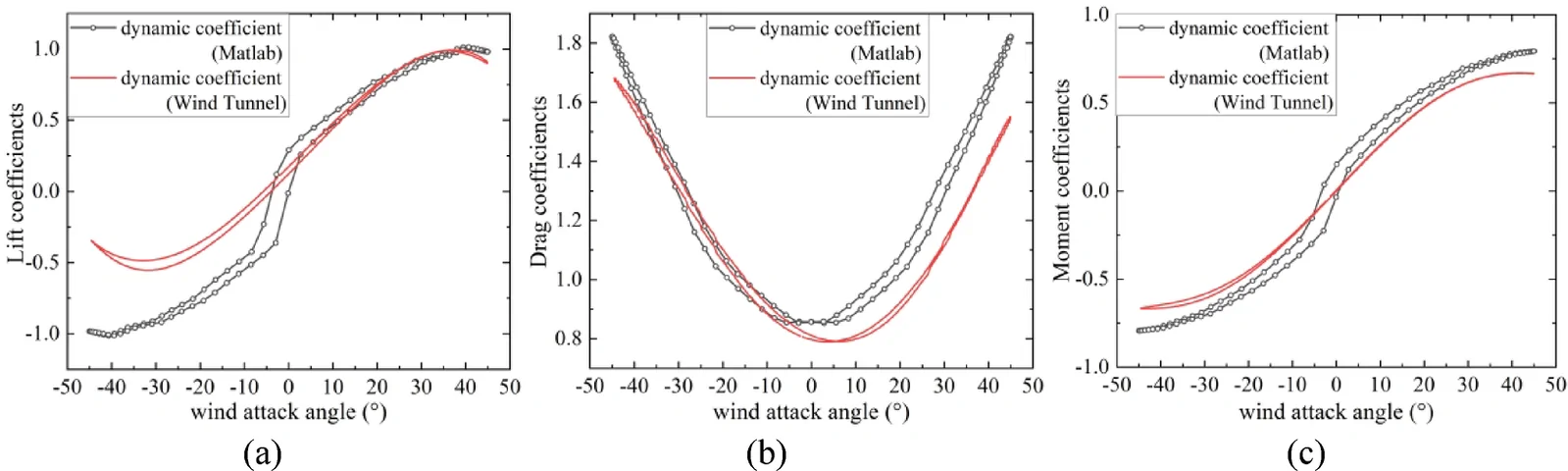
Comparison of unsteady aerodynamic coefficients by quasi-static assumption and wind tunnel test under wind velocity of 10 m/s and 14 mm thickness ice. (**a**) lift coefficients; (**b**) drag coefficients; (**c**) moment coefficients.

The unsteady aerodynamic coefficients curves of the crescent-shaped iced conductor based on the quasi-static assumption and the wind tunnel test have the same basic characteristics and obvious differences, as shown in Fig. 12. Figure 12a shows the unsteady aerodynamic coefficients curve based on quasi-static assumption and wind tunnel test with ice thickness 14 mm and wind velocity 10 m/s. It is seen that the curves of lift coefficients determined by the two methods are similar. At the wind attack angles 0° to 45°, the curve is basically the same. When the wind attack angle is less than 0°, the aerodynamic coefficients of the two curves at the same wind attack angle show a significant difference that the lift coefficients based on the quasi-static assumption are smaller than the results of the wind tunnel test. The drag coefficients shown in Fig. 12b is almost the same in the range of -45°~0°; when the wind attack angle is greater than 0°, the drag coefficients based on the quasi-static assumption are greater than the results of the wind tunnel test, and the peaks on both sides of the drag coefficients curve are also larger. Figure 12c shows that the moment coefficients of the two methods have relatively small differences in the range of -45°~45° wind attack angle. Despite these varying degrees of quantitative differences across the lift, drag, and moment coefficients, it is crucial to note that the overall changing trends and stability characteristics of each coefficient are highly similar in both cases. This similarity forms the fundamental rationale supporting the application of the quasi-static assumption in transmission line galloping analysis ^29–32^. As analyzed in Sect.  5.1, the observed differences between the two curves in Fig. 12 are also reflected in the dynamic and static aerodynamic coefficients from the wind tunnel test, which indirectly confirms the accuracy of the numerical model’s results.

In addition, the area of the curves based on the quasi-static assumption is generally larger, indicating that the aerodynamic forces do more work in this case. And compared to the smooth curves of the wind tunnel test, the curves based on the quasi-static assumption have more local slope increase, which is mainly reflected in the test process of the unsteady aerodynamic coefficients is continuous while the measurement of steady aerodynamic coefficients is a discontinuous process, which is continued by unsteady aerodynamic coefficient curves based on quasi-static assumption. From the analysis of the unsteady aerodynamic coefficient curve in the above chapter, it can be seen that the positive and negative of the main part of the work done by the aerodynamic force on the system is always consistent in the unsteady aerodynamic coefficients curve based on the quasi-static assumption and the wind tunnel test. In both cases, the area size of the main part of the work done by aerodynamic force with the changes of torsion frequency, wind velocity and ice thickness are also consistent, which also indicates that the quasi-static assumption has a certain applicability.

## Treatment of aerodynamic coefficients by polynomial fitting method

In fact, there are always measurement errors in wind tunnel tests, resulting in varying degrees of fluctuation in the measurement results and the dispersion of steady aerodynamic coefficients measured by wind tunnel tests is relatively large. Hence, some scholars^26,27^ perform polynomial fitting on aerodynamic coefficients before using them. This method can reduce the errors of aerodynamic coefficients obtained from wind tunnel tests to a certain extent. Therefore, the polynomial fitting method is also used to process the steady aerodynamic coefficients obtained from wind tunnel test in this paper, and analyzes the influence of selection of fitting order on the fitting results. Next, the steady aerodynamic coefficients after polynomial fitting were converted into unsteady aerodynamic coefficients, and the differences of unsteady aerodynamic coefficients under different fitting orders were compared, and finally compared with the wind tunnel test results.

**Fig. 13:**
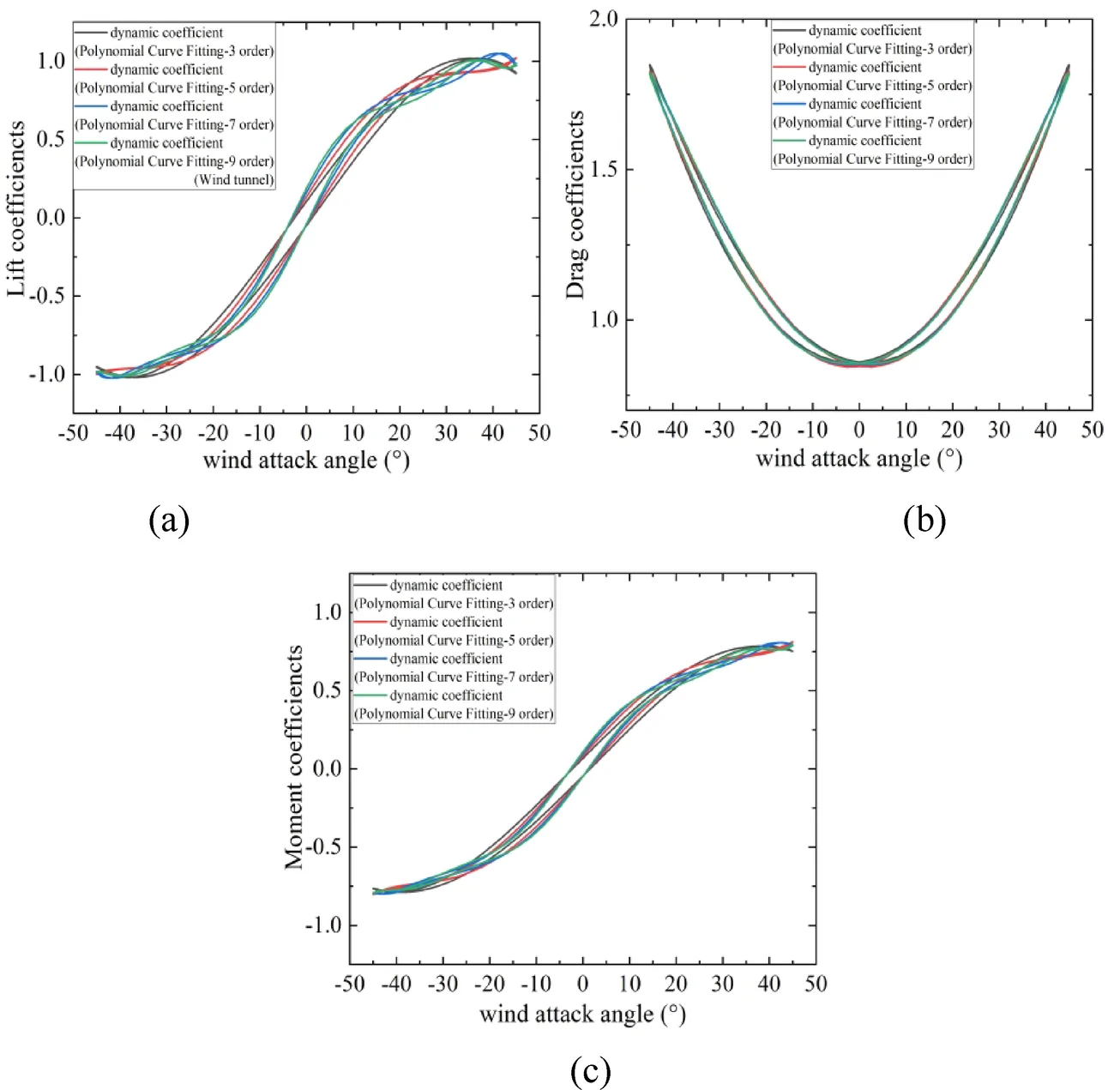
Comparison of steady aerodynamic coefficients of iced conductors and fitting results under different fitting orders at pitching frequency of 0.1 Hz, ice thickness of 14 mm and wind velocity of 10 m/s. (**a**) lift coefficients; (**b**) drag coefficients; (**c**) moment coefficients.

Because the existing research data does not clearly give the appropriate angle of attack range for the polynomial fitting processing of aerodynamic coefficients, and scholars generally choose a smaller angle of attack for polynomial fitting. Therefore, this paper considers that the fitting interval corresponds to the angle of attack range of the unsteady aerodynamic coefficient in the wind tunnel test, and chooses the fitting interval of -45°~45°. The comparison between the steady aerodynamic coefficients and the polynomial fitting results is shown in Fig. 13, where Fig. 13a, b and c respectively show the fitting results of the lift, drag and moment coefficients at angles of attack of -45°~45°, and the fitting order is 3 order and 9 order. The specific fitting formulas are as follows:

1.1 $${C_i}={a_0}+{a_1}\alpha +{a_2}{\alpha ^2}+ \cdots +{a_n}{\alpha ^n}{\text{ }}(i=L,D,M)$$

In the above equation, *a*_*n*_ represents the polynomial coefficients, and *α* is the wind attack angle. When *n* takes values of 3, 5, and 9, the equation represents 3rd-order, 5th-order, and 9th-order polynomial fittings, respectively.

**Fig. 14:**
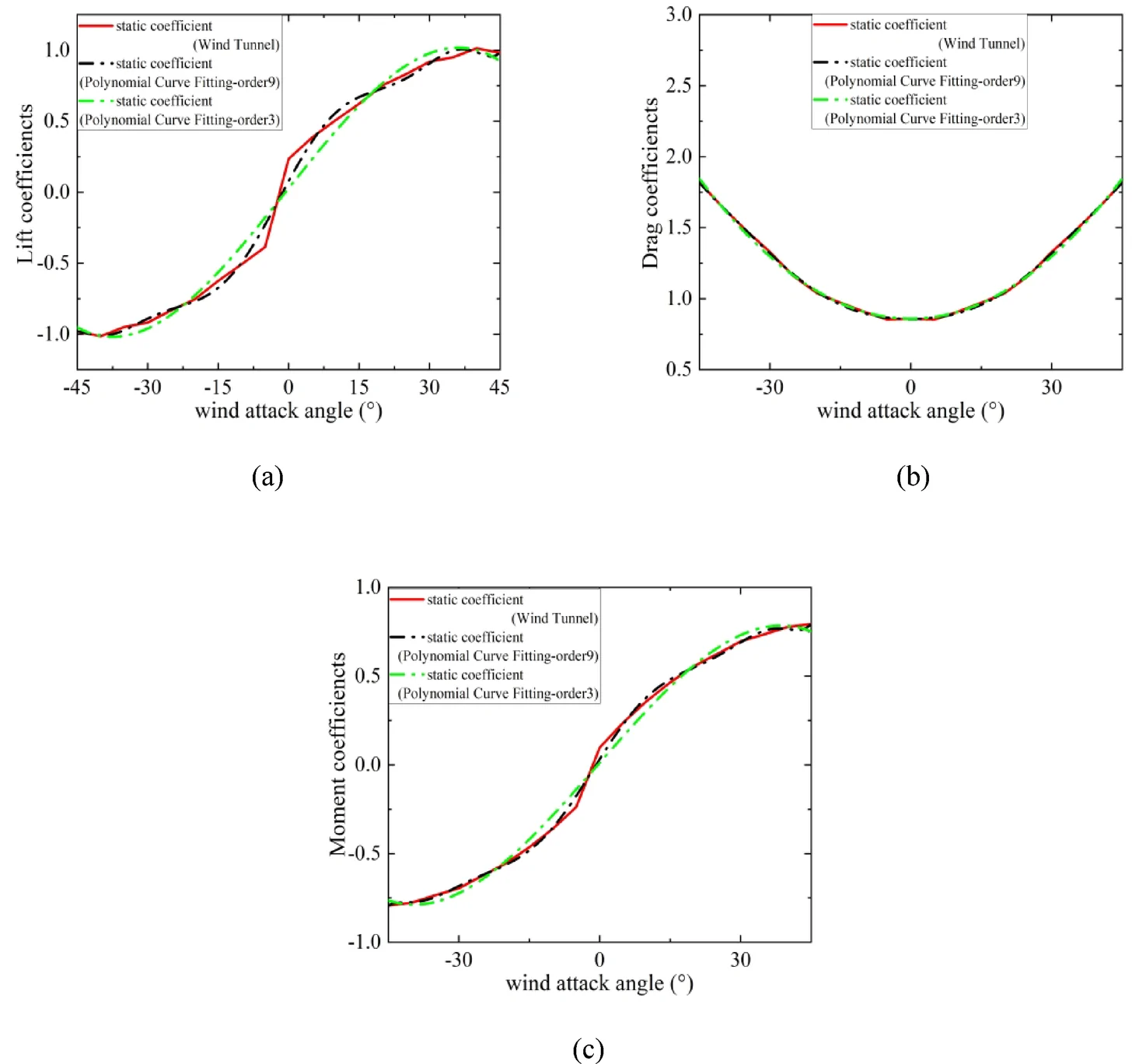
Comparison of unsteady aerodynamic coefficients of iced conductor varying with wind attack angle under different fitting orders at pitching frequency of 0.1 Hz, ice thickness of 14 mm and wind velocity of 10 m/s. (**a**) lift coefficients; (**b**) drag coefficients; (**c**) moment coefficients.

In order to further analyze the rationality of the selection of fitting order, the steady aerodynamic coefficients obtained by polynomial fitting are converted into unsteady aerodynamic coefficients, and the unsteady aerodynamic coefficients under different fitting orders are compared, in which the fitting orders are 3rd, 5th, 7th and 9th order, as shown in Fig. 14. It can be seen that the three unsteady aerodynamic coefficients have no obvious differences as a whole, and the drag coefficients are almost the same. With the increase of fitting order, the less the area that the lift coefficient and the moment coefficient curve envelop each other, and the curve with the fitting order of three or more have varying degrees of fluctuation between − 45°~-20° and 15°~45°. In order to further analyze the influence of the fitting order on the unsteady aerodynamic coefficients, the magnitude of the work done by each aerodynamic force to torsion is calculated separately, as shown in Table 5 (Since the lift, drag and moment coefficients mainly contribute to torsion work respectively: positive-negative-positive, and the area of the small loop included in the curve is small and does not affect the overall work, so only the area of the main work part is calculated).

From the data given in Table 8, it can be seen that the area of the main work part done by the aerodynamic force in the lift, drag and moment coefficients does not increase or decrease with the increase of the fitting order, and they do not show obvious regularity. Under different fitting orders, the difference between the size of work done by the aerodynamic force is comparatively large, while the moment coefficient is more stable, but the errors are all within 5%. Among them, the area of positive work in the lift and moment coefficients is the maximum when the fitting order is the 3rd order, and the negative work area in the drag coefficient is the minimum at the third order. Because of the great harm of conductor galloping to the transmission system, it is better to consider the most dangerous working conditions when choosing a reasonable fitting order, that is, the most prone to galloping. When the fitting order is 3th order, the positive work done by the aerodynamic force is the largest and the negative work is the smallest. At this time, the energy input into the system is the most, which makes it easier to cause the conductor to galloping compared with other working conditions. In addition, when performing galloping analysis, choosing a larger fitting order is also easy to lead to the increase of subsequent calculation, so it is appropriate to choose the third-order fitting.

**Table 8:** The area of the hysteresis loops of the aerodynamic coefficients under different fitting orders at pitching frequency of 0.1 Hz and ice thickness of 14 mm (MATLAB).

Fitting order	Lift (Positive work)	Drag (Negative work)	Moment (Positive work)
3	6.8268	2.1881	5.1916
5	6.6617	2.2446	0.0297
7	6.7939	2.2231	5.1803
9	6.7353	2.2060	5.1205

## BP (Back Propagation) neural network predicts aerodynamic coefficients

In the past, almost all the aerodynamic coefficients of iced transmission lines were obtained through wind tunnel tests and numerical simulation methods. However, wind tunnel tests have the limitations of high cost and time-consuming, and numerical methods are also prone to non-convergence. In this paper, the BP neural network method is used to predict the aerodynamic coefficients of transmission lines in different types of ice and different wind velocities, and compare them with results of the wind tunnel test.

### Database establishment

The aerodynamic coefficients of conductors with ice thicknesses of 14 mm, 24 mm, and 33 mm measured by wind tunnel tests at wind velocities of 10, 12, 14, 18 m/s are selected as the database of the BP neural network model. First, a part of wind tunnel test data was selected as the train set, and the other part as the test set; Then, the train set is imported into the model training, and the test set is imported after the prediction effect is feasible. Finally, the prediction data is generated and compared with the test set to observe the accuracy of the model.

### BP neural network model construction and parameter setting

The BP neural network (Backpropagation Neural Network), where “BP” explicitly stands for Backpropagation, is a widely used multilayer feedforward network. It is composed of a multilayer perceptron and the error backpropagation algorithm, possessing strong nonlinear mapping capabilities, generalization ability, and fault tolerance ^[28]^.

**Fig. 15:**
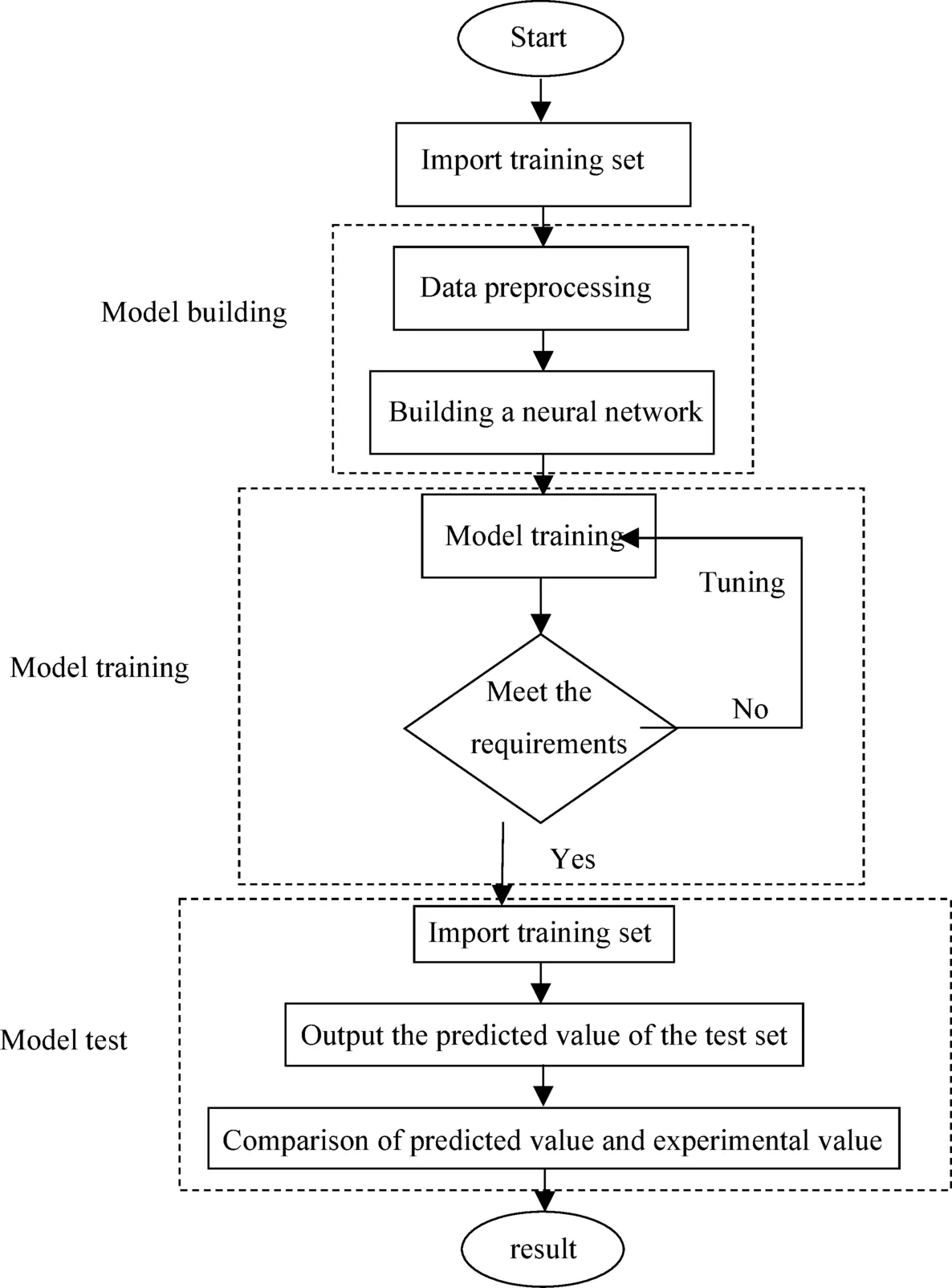
Flow chart of BP neural network.

A BP neural network typically consists of an input layer, at least one hidden layer, and an output layer. Its operational mechanism involves two core stages: the forward propagation of signals and the backpropagation of errors. In forward propagation, samples are input from the input layer, processed layer by layer through the hidden layer(s), and finally outputted as a prediction from the output layer. If a significant error exists between the predicted result of the output layer and the actual result, the process enters the error backpropagation phase. During this stage, the error is distributed to the neurons in each hidden layer according to the chain rule, and the weights (W) and biases (b) of these neurons are corrected using a gradient descent method to minimize the overall error, thereby making the output values more consistent with the actual values. Therefore, ensuring a sufficient number of neural units in the hidden layer can, to a certain extent, simulate arbitrarily complex nonlinear mappings. When the mapping ability is insufficient, it can be enhanced by adding a new hidden layer or increasing the number of neurons in existing hidden layers. In this paper, a multilayer BP neural network model is selected. Its algorithm flow chart is shown in Fig. 15.

**Fig. 16:**
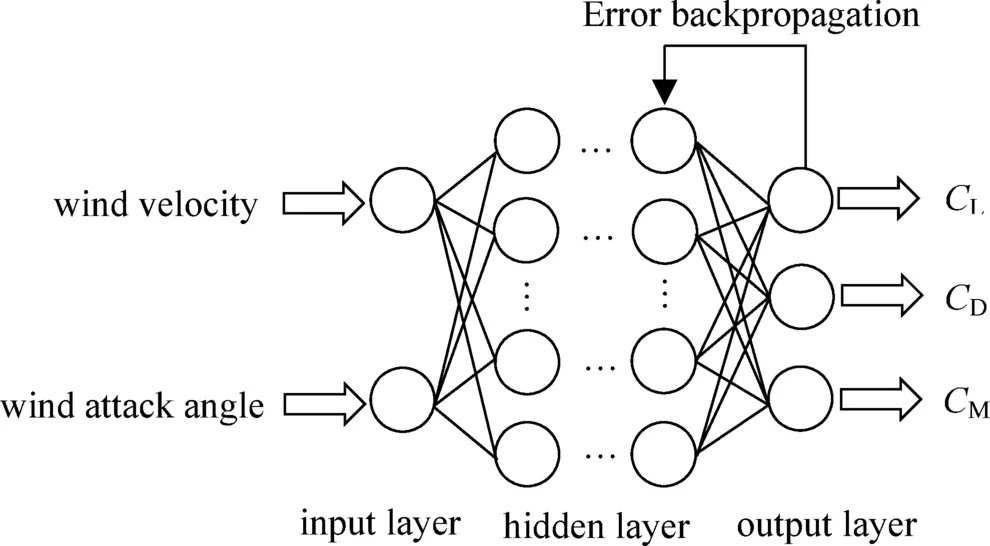
Frame diagram of multi-layer BP neural network.

According to the analysis of wind tunnel test results in Sect.  3, wind velocity and angle of attack, which affect the aerodynamic coefficients, are selected as the input parameters of the model, and the corresponding lift coefficients, drag coefficients, and moment coefficients are used as output parameters. These test data constitute the training set for the BP neural network. The model framework is shown in Fig. 16.

In the BP neural network model, due to the inconsistent input data units and different input data types, etc. It will lead to poor model convergence, slow training speed, and poor prediction effect. Therefore, the input data needs to be normalized before the model training, so that the input data is converted to the data type required by the model. In this paper, the BP neural network mainly uses trial and error method to adjust the parameters of the model. Specifically, the optimized network architecture consists of an input layer (wind velocity, attack angle), two hidden layers with 8 neurons each (8 × 8 structure), and an output layer (*C*_*L*_, *C*_*D*_, *C*_*M*_). This structure was selected based on a comparative analysis of convergence performance, where the 8 × 8 configuration minimized the Loss function more effectively than larger structures (e.g.,10 × 8 or 10 × 16) which showed signs of overfitting. The network employs the ReLU activation function and the Adam optimizer with a learning rate of 0.001. To ensure generalization and strictly control overfitting, the experimental dataset was randomly partitioned into a training set (70%), a validation set (15%), and a test set (15%). Furthermore, robust training strategies were employed, including Early Stopping (halting training when validation loss stagnates), L2 Regularization, and the injection of Gaussian noise (*N*(0,0.001)) into the training data, which acts as data augmentation to significantly enhance the model’s resilience against measurement uncertainties.”

### Prediction of aerodynamic coefficients

Taking the single conductor with the same ice thickness as the research object, predict the aerodynamic coefficients of the conductor under different wind velocities. Input variables: wind velocity, wind angle of attack; output variables: *C*_L_, *C*_D_, *C*_M_. The detailed working conditions are as follows:

1. Single conductor with ice thickness of 14 mm.
  ① The aerodynamic coefficients of 10, 12 and 14 m/s wind velocity predict the aerodynamic coefficients of 18 m/s wind velocity;
  ② The aerodynamic coefficients of 10, 12 and 18 m/s wind velocity predict the aerodynamic coefficients of 14 m/s wind velocity;
  ③ The aerodynamic coefficients of 10, 14 and 18 m/s wind velocity predict the aerodynamic coefficients of 12 m/s wind velocity.
  ④ The aerodynamic coefficients of 12, 14 and 18 m/s wind velocity predict the aerodynamic coefficients of 10 m/s wind velocity.
2. Single conductor with ice thickness of 24 mm.
  ① The aerodynamic coefficients of 10, 12 and 14 m/s wind velocity predict the aerodynamic coefficients of 18 m/s wind velocity;
  ② The aerodynamic coefficients of 10, 12 and 18 m/s wind velocity predict the aerodynamic coefficients of 14 m/s wind velocity;
  ③ The aerodynamic coefficients of 10, 14 and 18 m/s wind velocity predict the aerodynamic coefficients of 12 m/s wind velocity.
  ④ The aerodynamic coefficients of 12, 14 and 18 m/s wind velocity predict the aerodynamic coefficients of 10 m/s wind velocity.
3. Single conductor with ice thickness of 33 mm.
  ① The aerodynamic coefficients of 10, 12 and 14 m/s wind velocity predict the aerodynamic coefficients of 18 m/s wind velocity;
  ② The aerodynamic coefficients of 10, 12 and 18 m/s wind velocity predict the aerodynamic coefficients of 14 m/s wind velocity;
  ③ The aerodynamic coefficients of 10, 14 and 18 m/s wind velocity predict the aerodynamic coefficients of 12 m/s wind velocity.
  ④ The aerodynamic coefficients of 12, 14 and 18 m/s wind velocity predict the aerodynamic coefficients of 10 m/s wind velocity.

**Fig. 17:**
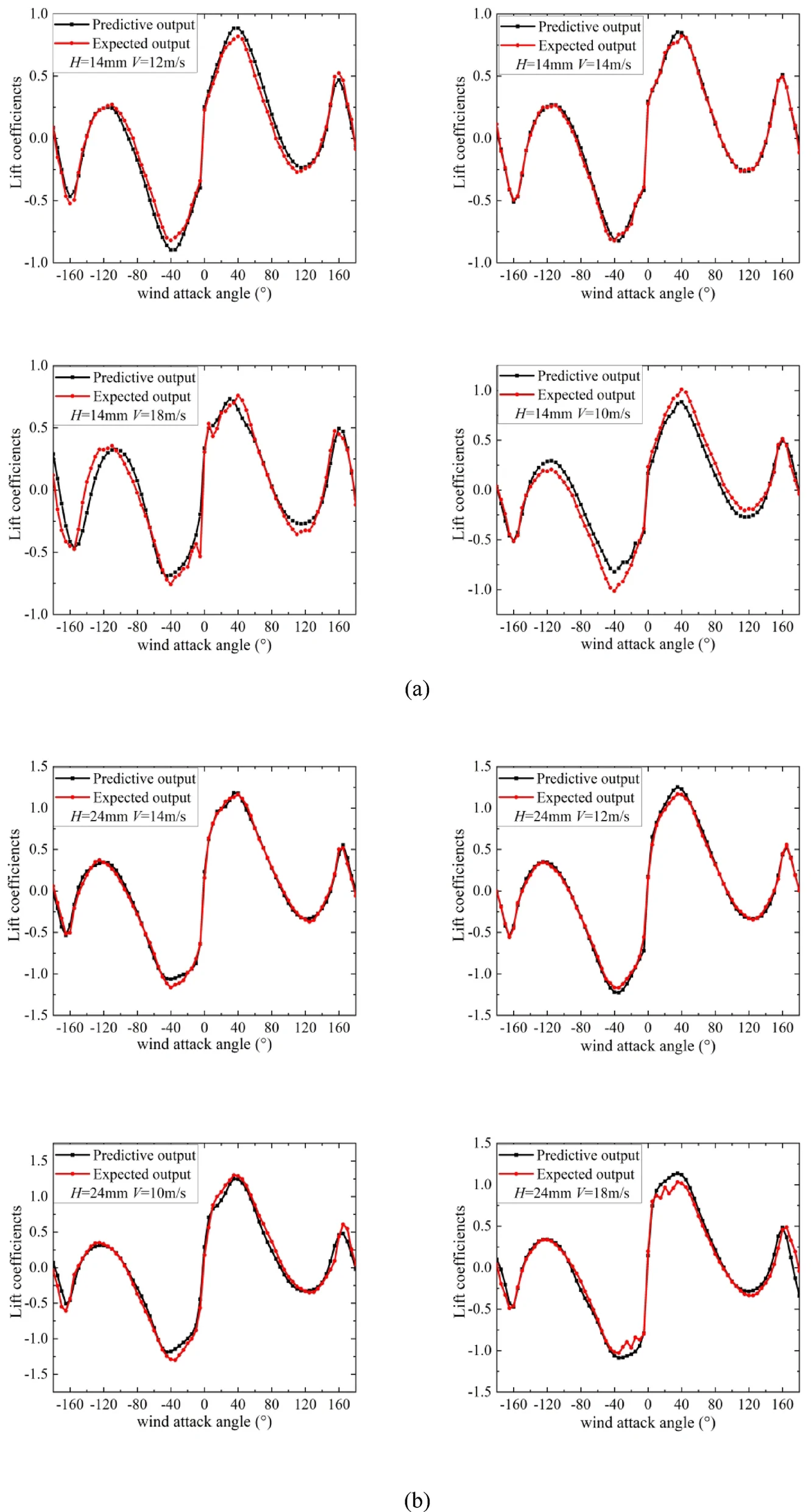

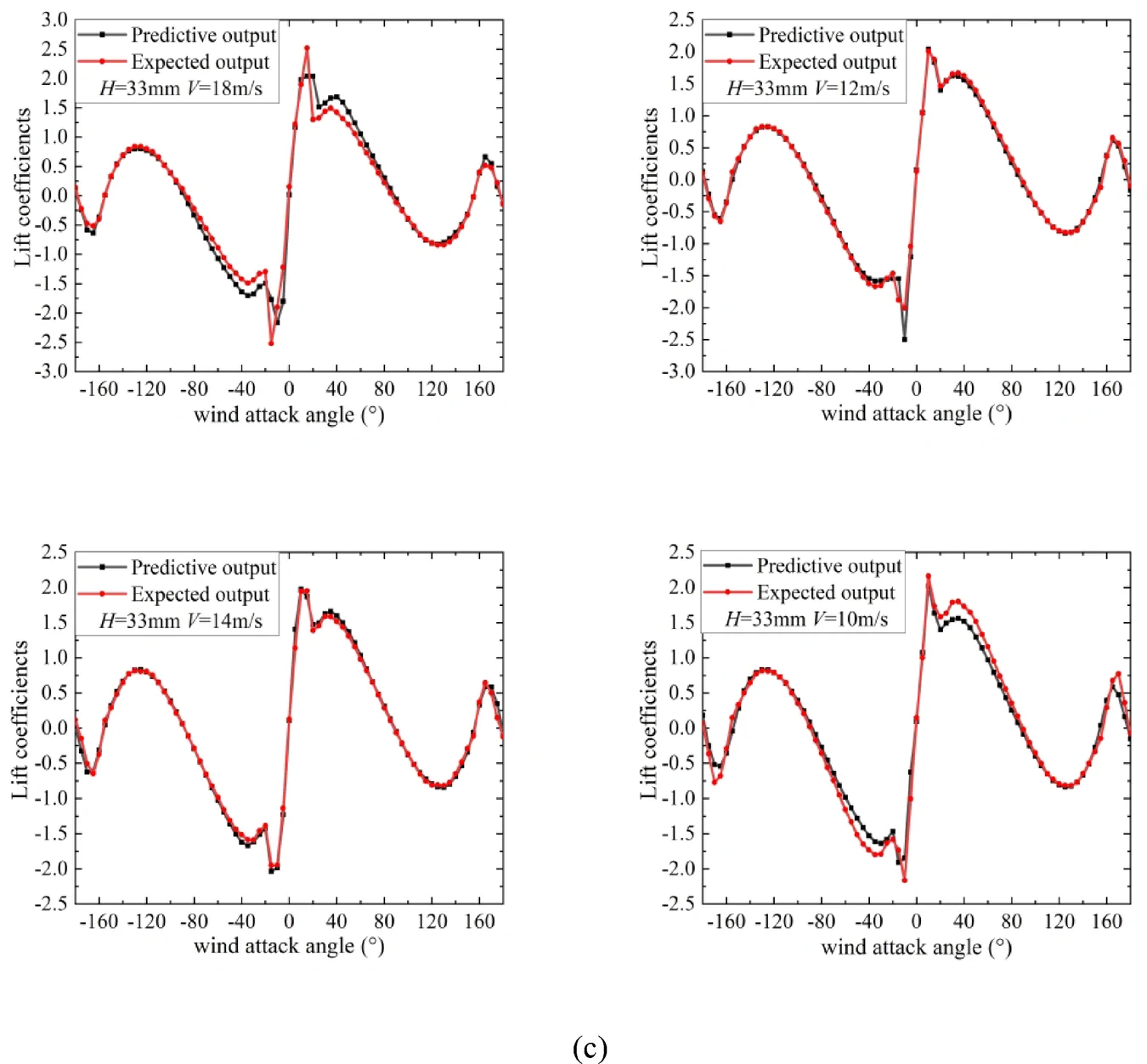
Prediction results of steady lift coefficients of the iced conductor under different ice thickness: (**a**) Ice thickness: 14 mm; (**b**) ice thickness: 24 mm and (**c**) ice thickness: 33 mm.

**Fig. 18:**
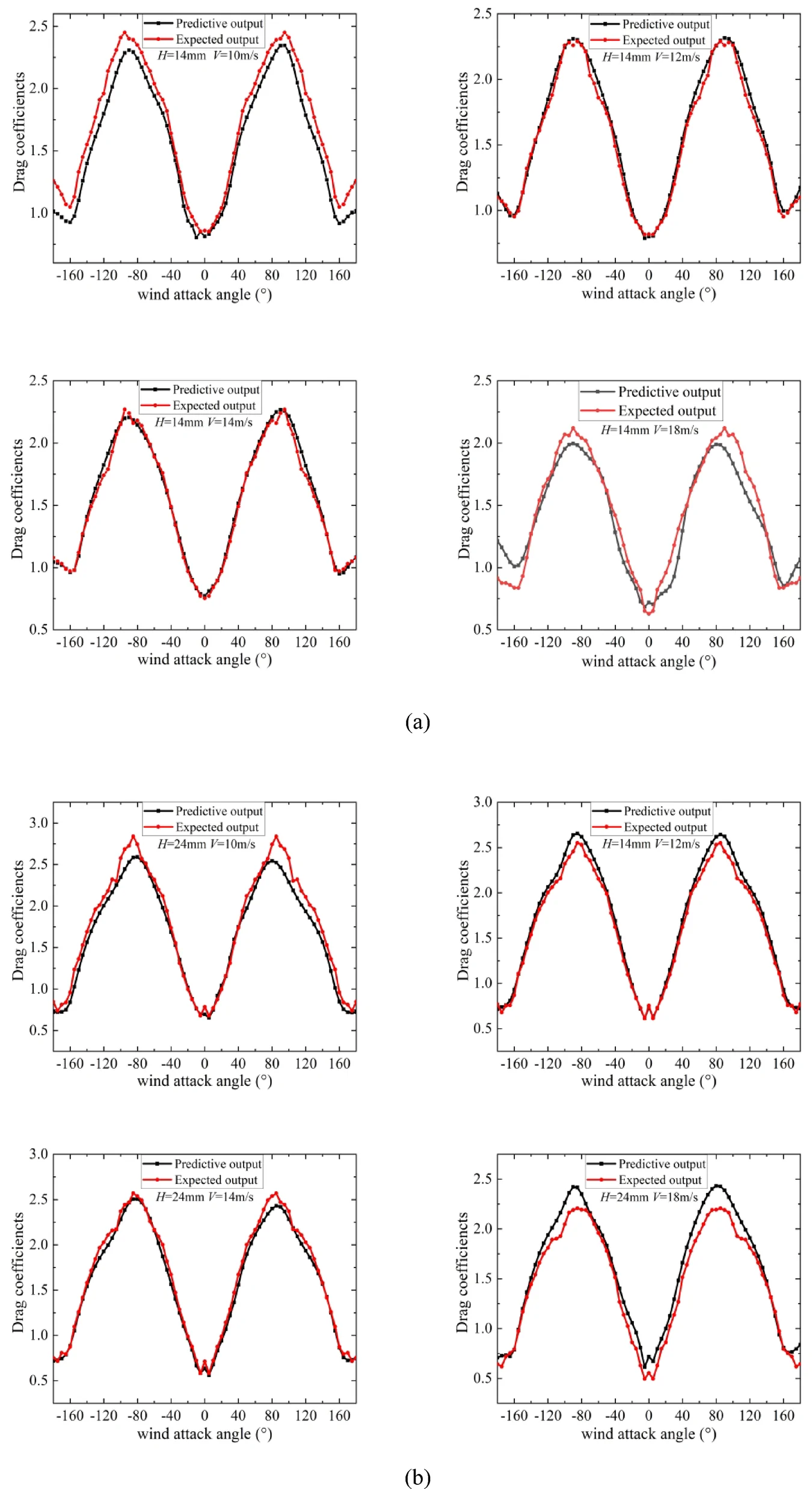

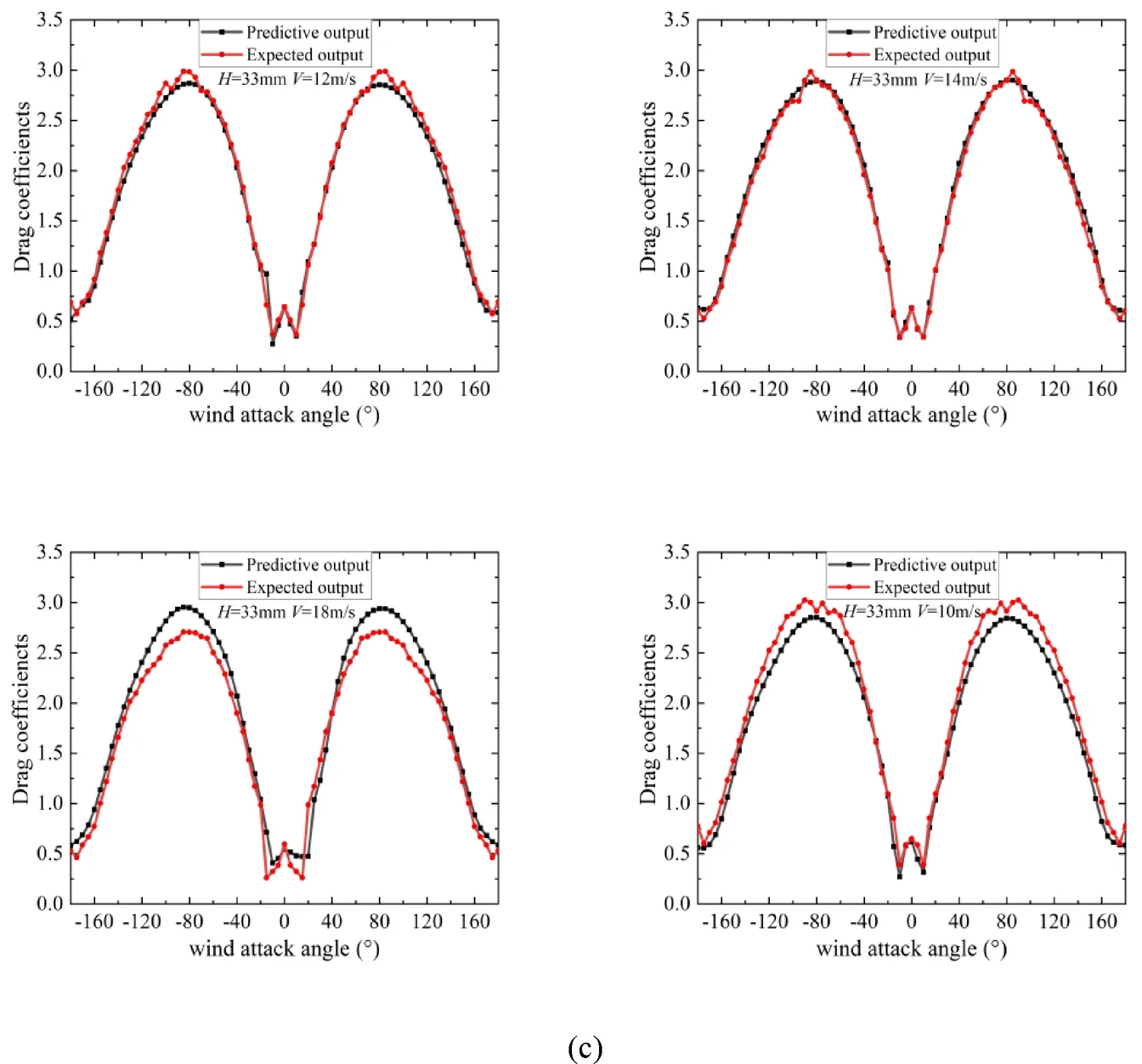
Prediction results of steady drag coefficients of the iced conductor under different ice thickness: (**a**) Ice thickness: 14 mm; (**b**) ice thickness: 24 mm and (**c**) ice thickness: 33 mm.

**Fig. 19:**
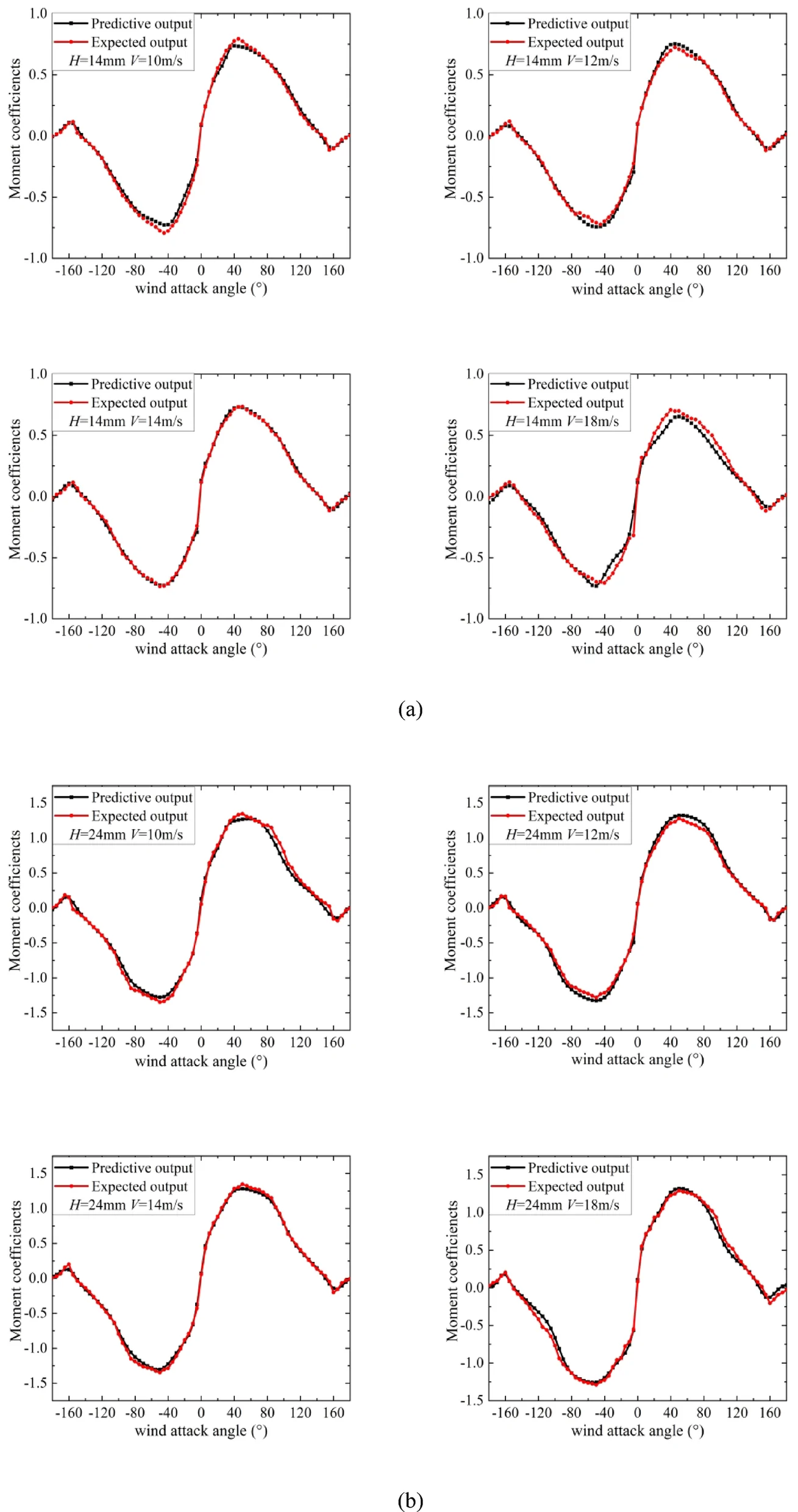

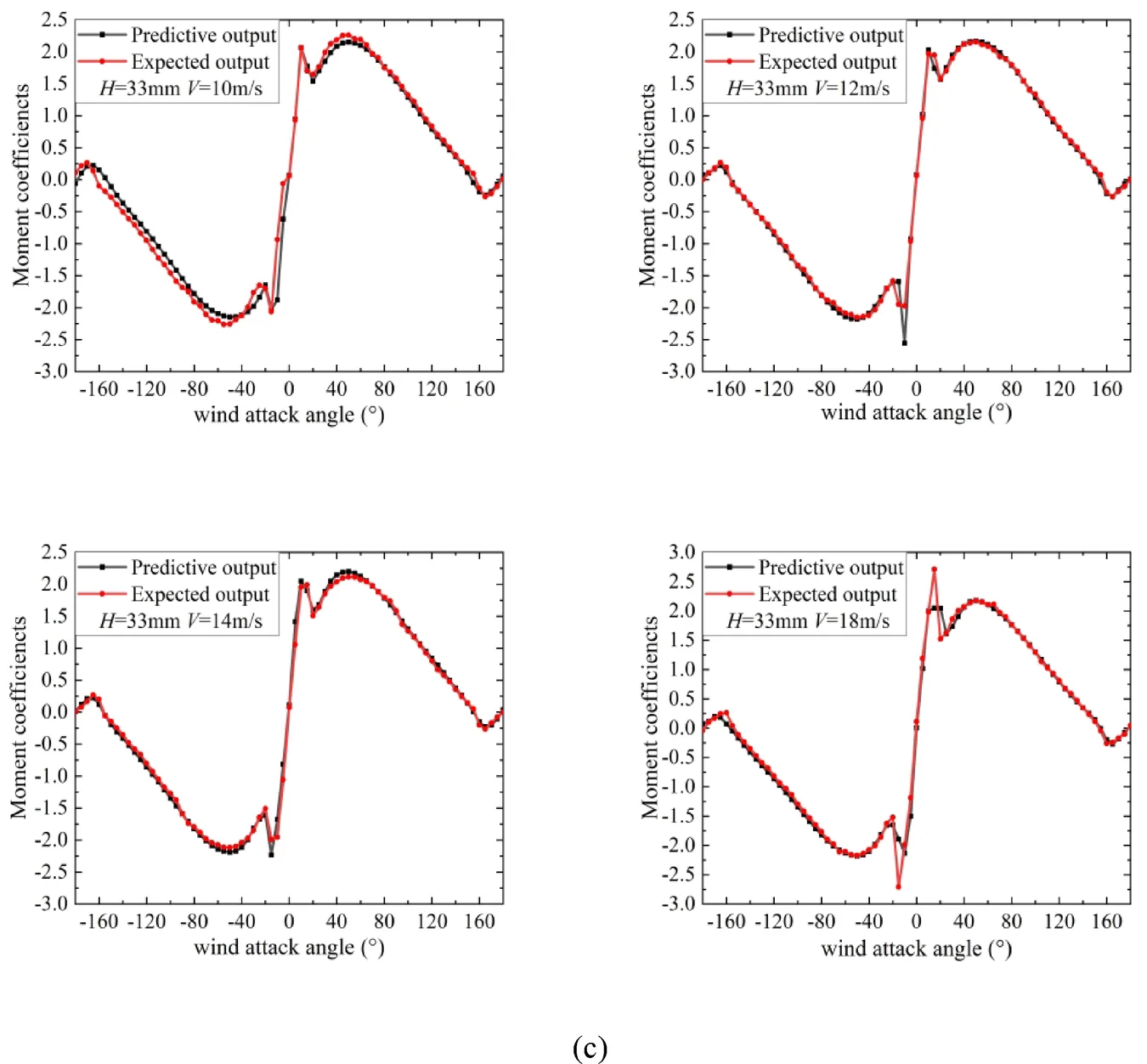
Prediction results of steady moment coefficients of the iced conductor under different ice thickness: (**a**) Ice thickness: 14 mm; (**b**) ice thickness: 24 mm and (**c**) ice thickness: 33 mm.

From the prediction results given in Figs. 17 and 18, and Fig. 19, it can be seen that the predicted output of steady aerodynamic coefficients is relatively close to the expected output, indicating that the neural network can reflect the potential law between the input data and the expected output to a certain extent. However, when the wind velocity is 10 m/s and 18 m/s, the aerodynamic coefficients have a larger relative error, while the prediction effect is better when wind velocity is 12 m/s and 14 m/s. For example: Fig. 17a shows that the lift coefficients have abrupt changes at -120°, -40° and 40° wind attack angle under wind velocity of 10 m/s. Similarly, when the wind velocity is 18 m/s, the lift coefficients at the corresponding wind attack angle also have difference. When the wind velocities are 10 m/s and 18 m/s, the drag coefficients under different ice thickness also have large relative error at the two peaks of -80° and 80°. Through the study of the error distribution trend, it is found that this is because the input wind velocity span is large and the input variables are not within the input variable interval of the original model. Although the prediction results are linearly similar, large errors are prone to occur. While predicting within the input wind speed range, the prediction ability is stronger and the effect is better. At the same time, when the wind velocity is high (18 m/s), several abnormal points appear in the test results, which lead to the unobvious potential law of the sample data distribution, and make the neural network recognition effect is not good, which also leads to a relatively large error. In general, compared with drag coefficients and lift coefficients, the prediction effect of moment coefficients is better, mainly because the distribution law of moment coefficients itself is better, and the sample data is stable and the fluctuation is small, and the regularity of original data directly determines whether the neural network can obtain good learning effect.

### Error analysis and model validation

Based on the prediction models constructed using the BP neural network, we performed a statistical analysis of the coefficient of determination (R^2^) for lift, drag, and torque coefficients under different ice thicknesses (14 mm, 24 mm, 33 mm) and wind speeds. The statistical results indicate that the model possesses excellent overall fitting performance. The calculated R^2^ values for the vast majority of working conditions exceed 0.95, with some cases approaching 0.999. This quantitative evidence demonstrates that the neural network effectively captures the nonlinear data variation laws observed in wind tunnel experiments.

Even as the ice thickness increases from 14 mm to 33 mm, the model maintains high fitting stability. A comparison between the predicted values and actual wind tunnel experimental data reveals that the prediction points are tightly distributed around the ideal fit line y = x, showing a significant linear clustering trend without obvious systematic bias. Specifically, the torque coefficient predictions are the most robust, fitting closely to the experimental values even at the extremes of the coefficient range. While the lift and drag coefficients show slight dispersion in high-value regions, the overall error remains within an acceptable range for engineering applications.

## Comparison of all results

**Fig. 20:**
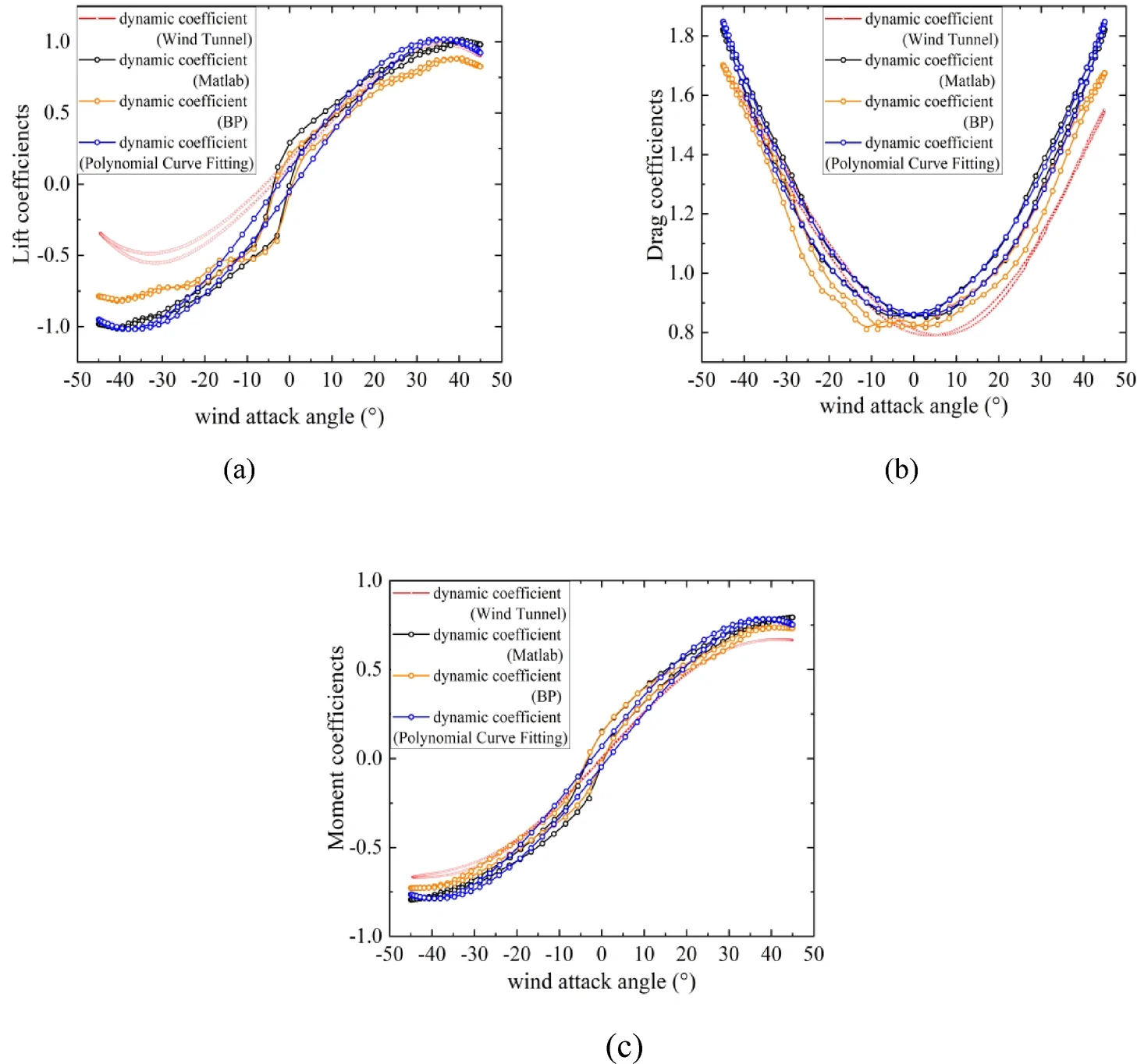
Comparison of the unsteady aerodynamic coefficients of conductors under wind velocity of 10 m/s and ice thickness of 14 mm. (**a**) lift coefficients; (**b**) drag coefficients; (**c**) moment coefficients.

The comparison of the unsteady aerodynamic coefficients of conductors under wind velocity of 10 m/s and ice thickness of 14 mm obtained from wind tunnel test and various numerical methods is shown in Fig. 20, in which the results of the numerical simulation can be divided into three working conditions according to the different processing methods of the basic sample data: (1) wind tunnel test data; (2) BP neural network prediction; (3) polynomial fitting. It can be seen that although there are differences in the values of the four curves, the changing laws of the lift, drag and moment coefficients determined by the numerical simulation and the wind tunnel tests are consistent. From the comparison of the unsteady lift coefficients shown in Fig. 20a, it can be seen that the area of loop represents the positive work done by aerodynamic lift is larger than the negative work part in the four curves. From the point of view of the curve area alone, the curve area of the wind tunnel test is the smallest. The aerodynamic coefficients curve predicted by BP neural network is consistent with the untreated curve, but there is a certain degree of deviation on the left and right side, and there are many small fluctuations in the value. This difference may be caused by the error between the predicted output and the expected output of the BP neural network (as shown in figure… it can be seen that there is a large relative error between the predicted output and the expected output at wind velocity of 10 m/s, and there is numerical fluctuation at the curve corner). At the same time, the unsteady aerodynamic coefficients obtained by polynomial fitting process are smoother on the whole, without the phenomenon of local curvature increase, and have a higher agreement with the results of wind tunnel test. As for the drag coefficients, the aerodynamic coefficients curve predicted by BP neural network also has phenomenon of deviation and numerical fluctuation, which may also be due to the relative error between the predicted output and the expected output. In addition to results of the wind tunnel test, the positive and negative areas in the remaining three curves are basically the same, which is related to the fact that the numerical model does not consider the relative wind velocity. The four moment coefficients curves shown in Fig. 20c all shows high similarity, and the work done by aerodynamic force is mainly positive.

**Fig. 21:**
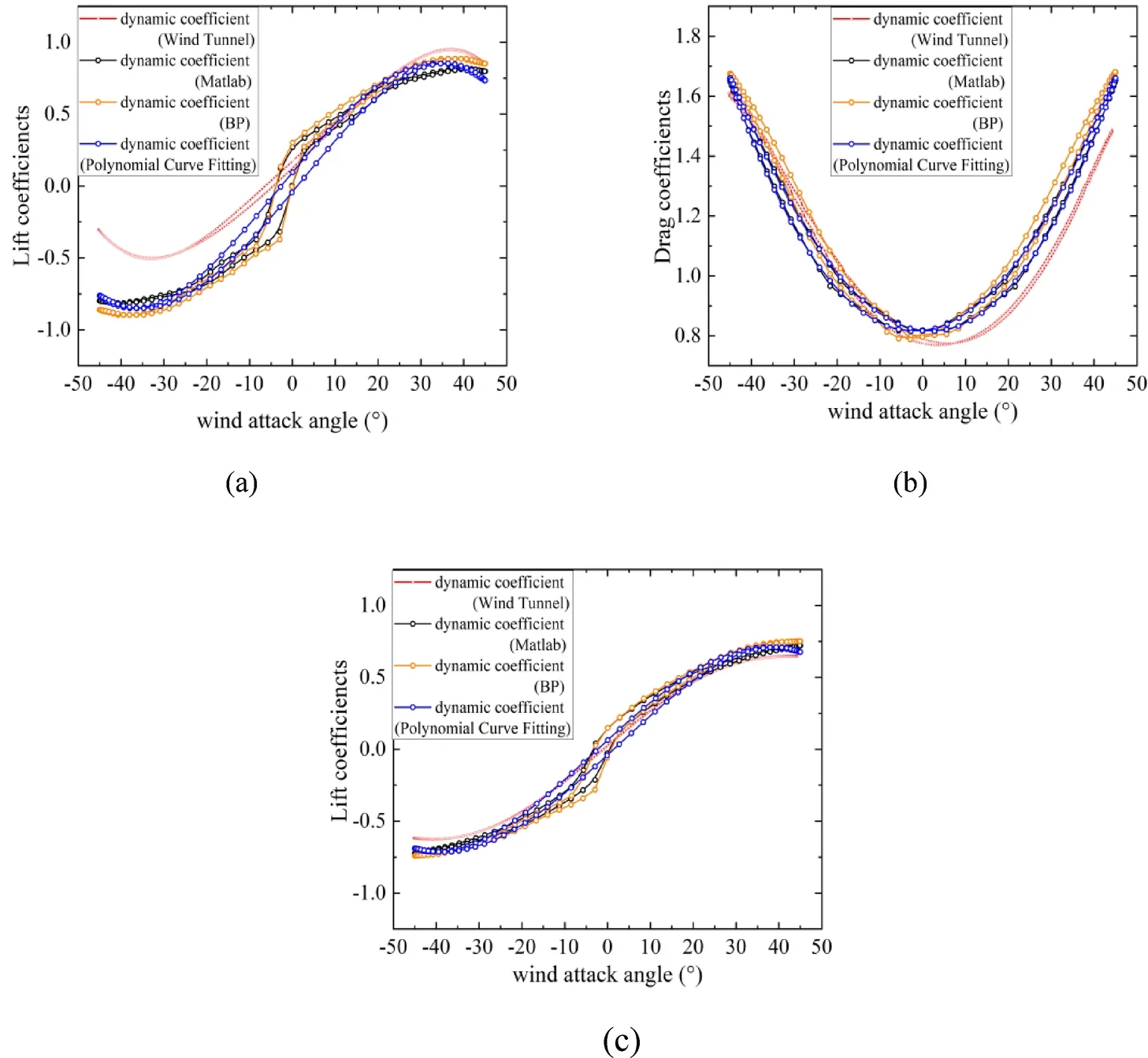
Comparison of the unsteady aerodynamic coefficients of conductors under wind velocity of 12 m/s and ice thickness of 14 mm. (**a**) lift coefficients; (**b**) drag coefficients; (**c**) moment coefficients.

Figure 21 shows the comparison of the unsteady aerodynamic coefficients of conductors under wind velocity of 12 m/s and ice thickness of 14 mm obtained from wind tunnel test and numerical simulation. It can be seen that the changing laws of lift, drag and moment coefficients are also consistent with the wind tunnel test, and the aerodynamic coefficients curve predicted by the BP neural network has little difference with the untreated curve. Meanwhile, from the prediction results of the BP neural network in Sect.  7.1 above (Figs. 16, 17 and 18), it can be seen that the prediction effect of the BP neural network is better at wind velocity of 12 m/s. Therefore, these phenomena indicate that the degree of agreement between the unsteady aerodynamic coefficients converted from the **s**teady aerodynamic coefficients predicted by the BP neural network as the sample data and the wind tunnel test results depends on the quality of the prediction effect.

Based on the above results, it can be seen that no matter which approach is adopted, the curves obtained are always consistent with the wind tunnel test results in terms of the positive and negative aspects of work done by the aerodynamic force, and the change law of the curve is the same, which proves the applicability of the quasi-static hypothesis. From the point of view of the curve area alone, the area of the unsteady aerodynamic coefficient curve obtained after polynomial fitting processing is the largest, while the area of the curve in the wind tunnel test is the smallest. From this point, it can be concluded that the aerodynamic coefficients obtained by the quasi-static hypothesis are more conservative in terms of work compared with the results of wind tunnel tests, and this phenomenon is further amplified after polynomial fitting processing.

## Conclusion

The steady and unsteady aerodynamic coefficients of the iced conductor are obtained by wind tunnel tests, the causes of the unsteady aerodynamic coefficients and the positive or negative work done by aerodynamic force represented by each loop are analyzed, and the influence of each parameter on the unsteady aerodynamic coefficients is studied. Based on the quasi-static hypothesis and through MATLAB programming, the conversion of steady aerodynamic coefficients to unsteady aerodynamic coefficients was realized. The applicability of the quasi-static assumptions was discussed by comparing it with the unsteady aerodynamic coefficients obtained by wind tunnel tests. Then, the polynomial fitting method is used to process the steady aerodynamic coefficients, and the influence of the selection of different fitting orders on work done by the aerodynamic force is discussed. In addition, the BP neural network method is used, a prediction model of the aerodynamic coefficients of the iced conductor is established, and the aerodynamic coefficients are predicted. Finally, the steady aerodynamic coefficients obtained by the above-mentioned different processing methods are converted into unsteady aerodynamic coefficients by MATLAB, and the comparison is made. It is concluded that:

1. The unsteady aerodynamic coefficients exhibit distinct hysteresis loops compared to the single-valued steady coefficients. This phenomenon is primarily driven by the time-lagged flow separation and reattachment on the iced surface: the boundary layer response cannot instantaneously follow the conductor’s rotation due to fluid inertia. Additionally, the geometric asymmetry of the crescent ice amplifies these non-linear unsteady effects. In terms of dynamics, this hysteresis creates a phase shift between force and displacement. The enclosed area of the hysteresis loop quantifies the aerodynamic work: a clockwise lift loop indicates positive energy input, which destabilizes the conductor and drives galloping, while a counter-clockwise loop provides aerodynamic damping. Despite these dynamic differences, the overall changing trends of steady and unsteady coefficients remain consistent, with the steady curves effectively representing the average value of the unsteady hysteresis loops.
2. Parameter studies reveal consistent evolutionary trends for both quasi-static and unsteady methods, despite their quantitative discrepancies. Specifically, the area of positive aerodynamic work is observed to decrease as the wind velocity increases, suggesting a potential reduction in galloping intensity at higher subcritical speeds. Conversely, the positive work area increases significantly with ice thickness, confirming that thicker ice accretion poses a higher instability risk. Furthermore, in terms of energy flow direction, both methods consistently identify aerodynamic lift and moment as the sources of positive work, while aerodynamic drag provides negative work.
3. The evolution laws of lift, drag, and moment coefficients for iced conductors obtained via the numerical quasi-static model are consistent with those measured in wind tunnel tests, although distinct limitations exist regarding quantitative applicability. In terms of aerodynamic work, the primary work-contributing intervals for lift, drag, and moment remain identical in both cases, indicating that the quasi-static hypothesis possesses a degree of validity. Although the experimental data density at high frequencies was constrained by the bin-sampling size, potentially affecting the reliability of local data, the high degree of correlation across parameter trends validates the fundamental applicability of the quasi-static hypothesis for this study.
4. Different fitting orders have an impact on the amount of aerodynamic work. After comprehensive consideration, it is more appropriate to choose the third-order polynomial fitting.
5. The aerodynamic coefficients predicted by BP neural network are close to the results of wind tunnel tests, which verifies the feasibility of this method. At the same time, it can be seen from the prediction results that the prediction ability is stronger and the effect is better in the prediction within the input wind velocity interval. The regularity of the original data directly determines whether the neural network can obtain a good learning effect.
6. Engineering Implications: The identified aerodynamic work characteristics provide a direct basis for risk assessment. The conditions exhibiting large positive work areas represent high-risk zones for galloping onset and fatigue damage. For utility operators, we recommend a two-tiered strategy: prioritizing anti-galloping devices like inter-phase spacers in regions prone to these meteorological conditions during the design phase, and calibrating operational monitoring systems to trigger early warnings when real-time data enters these high-energy input regimes.

## Supplementary Information

Below is the link to the electronic supplementary material.

Supplementary Material 1

## Data Availability

The datasets generated and analysed during the current study are available in the supplementary information files of this article. The raw data are provided in Origin Project format.
